# A new modular neuroprosthesis suitable for hybrid FES-robot applications and tailored assistance

**DOI:** 10.1186/s12984-024-01450-6

**Published:** 2024-09-04

**Authors:** Javier Gil-Castillo, Diana Herrera-Valenzuela, Diego Torricelli, Ángel Gil-Agudo, Eloy Opisso, Joan Vidal, Josep M. Font-Llagunes, Antonio J. del-Ama, Juan C. Moreno

**Affiliations:** 1 BioRobotics Group, Center for Automation and Robotics, CSIC, Madrid, Spain; 2 ETSI Telecomunicación, Universidad Politécnica de Madrid, Madrid, España; 3 International Doctoral School, Rey Juan Carlos University, Madrid, Spain; 4 Biomechanics and Technical Aids Unit, National Hospital for Paraplegics, Toledo, Spain; 5 Unit of Neurorehabilitation, Biomechanics and Sensorimotor Function (HNP-SESCAM), Associated Unit of R&D&I to the CSIC, Madrid, Spain; 6 Institut Guttmann, Institut Universitari de Neurorehabilitació adscrit a la UAB, Badalona, Barcelona, 08916 Spain; 7 Biomechanical Engineering Lab, Department of Mechanical Engineering and Research Centre for Biomedical Engineering, Universitat Politècnica de Catalunya, Diagonal 647, Barcelona, 08028 Spain; 8 Institut de Recerca Sant Joan de Déu, Santa Rosa 39-57, Esplugues de Llobregat, 08950 Spain; 9 Bioengineering Systems and Technologies Research Group, Department of Applied Mathematics, Materials Science and Engineering and Electronic Technology, Rey Juan Carlos University, C/ Tulipan S/N, Móstoles, 28933 Spain

**Keywords:** Modular systems, Neuroprostheses, Functional electrical stimulation, Wearable exoskeletons, Hybrid FES-exoskeleton, Personalization, Gait, Kinematics

## Abstract

**Background:**

To overcome the application limitations of functional electrical stimulation (FES), such as fatigue or nonlinear muscle response, the combination of neuroprosthetic systems with robotic devices has been evaluated, resulting in hybrid systems that have promising potential. However, current technology shows a lack of flexibility to adapt to the needs of any application, context or individual. The main objective of this study is the development of a new modular neuroprosthetic system suitable for hybrid FES-robot applications to meet these needs.

**Methods:**

In this study, we conducted an analysis of the requirements for developing hybrid FES-robot systems and reviewed existing literature on similar systems. Building upon these insights, we developed a novel modular neuroprosthetic system tailored for hybrid applications. The system was specifically adapted for gait assistance, and a technological personalization process based on clinical criteria was devised. This process was used to generate different system configurations adjusted to four individuals with spinal cord injury or stroke. The effect of each system configuration on gait kinematic metrics was analyzed by using repeated measures ANOVA or Friedman’s test.

**Results:**

A modular NP system has been developed that is distinguished by its flexibility, scalability and personalization capabilities. With excellent connection characteristics, it can be effectively integrated with robotic devices. Its 3D design facilitates fitting both as a stand-alone system and in combination with other robotic devices. In addition, it meets rigorous requirements for safe use by incorporating appropriate safety protocols, and features appropriate battery autonomy, weight and dimensions. Different technological configurations adapted to the needs of each patient were obtained, which demonstrated an impact on the kinematic gait pattern comparable to that of other devices reported in the literature.

**Conclusions:**

The system met the identified technical requirements, showcasing advancements compared to systems reported in the literature. In addition, it demonstrated its versatility and capacity to be combined with robotic devices forming hybrids, adapting well to the gait application. Moreover, the personalization procedure proved to be useful in obtaining various system configurations tailored to the diverse needs of individuals.

**Supplementary Information:**

The online version contains supplementary material available at 10.1186/s12984-024-01450-6.

## Background

Functional electrical stimulation (FES) is an effective technique to assist and rehabilitate functional movements due to neurological disorders [1–5]. However, uncertainties exist for the optimal application of FES adapted to the physiology of different users. The optimal settings of the parameters defining electrical stimulation (ES) are unknown, so the adjustment is often based on personal experience and generic protocols that are not personalized and often lack scientific evidence [6–8].

This technique is applied through neuroprostheses (NPs), whose general architecture is composed of sensors, a control unit (CU), an electrical stimulation unit or electrostimulator, and electrodes [1, 9, 10]. However, the optimal choice of sensor or combination of sensors is unclear and depends on the characteristics of the individual and the context of use [10–12]. Furthermore, electrostimulators are frequently designed with multiple channels to enable control of several muscle groups during an activity or to apply diverse strategies [10]. Nevertheless, the greater the number of channels, the higher the power required, resulting in bulky and heavy non-portable systems that often need to be connected to a power outlet [13–15]. As a result, we currently find non-flexible portable NP systems that are optimized for a specific application and non-portable systems that offer greater flexibility [15–18]. All this, together with the complexity and diversity of applications and target populations, the heterogeneity within the same population and even the variability of the target individual over time, results in a limited use of NPs when adapting to any individual, context or application [19].

Consequently, certain research groups have developed systems with modular or distributed architectures that add features of great importance to the general NP architecture [8, 15, 20–28]. Their flexible and scalable architecture allows the integration of a variable number of sensors of various types and the addition of more electrostimulators to expand the number of ES channels of the system. These electrostimulators are all portable, powered by built-in batteries, operate within the safety limits of ES, and typically employ transcutaneous electrodes. The connection between components is crucial and ensures proper system operation and they usually offer a graphical user interface (GUI) that, among other functionalities, allows configuration of the basic ES parameters (amplitude, pulse width and frequency) and pulse trains, which are usually trapezoidal.

These systems have represented a technological breakthrough, but the limitations of FES application are still evident. This has led to the development of hybrid systems where that integrate NPs with robotic assistive devices, aiming to leverage the strengths of both systems and mitigate their respective weaknesses [29–32]. Notably, none of the above NPs have been combined with a robotic device, and existing hybrid wearable systems in the literature tend to be specific to solve or study particular problems [29, 33]. Despite this, the potential of hybrid systems in rehabilitation is evident, offering benefits such as reduced user energy expenditure, delayed onset of muscle fatigue, and improved posture and movement stability [31, 34]. Nevertheless, while the hybrid approach holds promise, these systems are not yet mature enough for clinical trials to fully uncover their impact on end users [35].

The above highlights the urgency of creating modular NPs suitable for hybrid FES-robot applications that overcome current limitations and allow exploring more efficient assistance strategies. In this paper, we introduce an innovative modular NP that stands out for its flexibility, scalability, customizability, and ability to integrate robotic devices. We also present a personalization procedure designed to generate system configurations that are tailored to individual needs, with the aim of providing effective and appropriate assistance in a wide variety of contexts and applications. This publication is structured in five main parts. Firstly, the design requirements and the system and its characteristics are shown in detail. Secondly, to illustrate the flexibility of our device, we showcase a possible adaptation of the system for its application in gait rehabilitation, its combination with a lower limb wearable robot (WR) and a personalization procedure, enabling system adjustments tailored to individual needs based on clinical criteria. Thirdly, a validation test is conducted with four individuals who have experienced a stroke or spinal cord injury (SCI) in order to evaluate the versatility and personalization capacity of the system for different assistance needs. Fourthly, the technical results of the development are presented, highlighting ideal characteristics in terms of component integration, connection and communication capacity, simple and safe assembly with robotic devices, safety, appropriate dimensions and weight, as well as adequate functionalities for its implementation in different contexts. In addition, results are presented that support the versatility of the system when used with individuals with different needs, as well as the kinematic impact on the gait pattern of the configurations tested in each user. These results are comparable to those reported in the literature for other systems. Finally, an analysis of the results and limitations is carried out and future development perspectives are discussed.

## Methods

We present the identified requirements for designing a modular NP system suitable for hybrid FES-robot applications. Then we outline the development of such a system, the adaptation to gait rehabilitation to illustrate the capacity of the NP for personalizing assistance. Then, we present the experimental results showcasing tailored assistance provided to individuals with different functional needs.

### System requirements and design specifications

The new NP system presented here has been developed within the framework of the TAILOR Project (with reference RTI2018-097290-B-C31) under a multidisciplinary collaboration that aims to effectively address the diversity and uniqueness of the needs and preferences of motor NP users, contributing to the acceptance and successful integration of these systems in an infinite number of applications and contexts. To achieve this goal, literature was taken as a baseline and interdisciplinary meetings were held with engineers and physiotherapists, which allowed the establishment of a series of fundamental design requirements:

- Architecture: the system must offer a modular architecture that allows greater flexibility in the configuration and reconfiguration of hardware and software components. In this sense, attention should be paid to the flexibility for the integration and configuration of different types of sensors that allow the acquisition of the necessary information adapting to the application, context or individual capabilities. In addition, it must be scalable to be able to adjust the complexity and capabilities of the system without having to replace it completely. In other words, it must be able to grow or expand by incorporating new sensors or more stimulation channels without losing power in order to address more muscle groups, motor functions or apply specific stimulation strategies [10, 36, 37]. For this purpose, it is necessary to use a network topology where the CU serves as a communication interface with external systems and powerful electrostimulation nodes. These nodes, where the electrostimulation electronics are located, must have few channels and control the ES parameters precisely and reliably, in compliance with electrical and medical safety standards. Based on the literature, it is also preferable that the system be geared to the use of transcutaneous electrodes [1, 4, 38–43]. In addition, it should be a tailored system that facilitates the configuration of the electrostimulators and the parameters related to ES.
- Connection: effective connection between system components is crucial. Wireless connection should be favored to provide maximum flexibility and mobility, but latency and security must be considered. Real time or minimum latency must be guaranteed both in the reception of information from sensors and external systems and in the capacity to act. This makes it possible to provide instant feedback and a fast and accurate response so that the assistance can be effective in any application and context, achieving coordinated and natural movements. All this goes through the selection of a CU capable of providing and managing these interactions through efficient connection interfaces.
- Neuro-robotic integration: the system must be able to interconnect with robotic devices in order to improve its functionality and usefulness by offering a more complete and versatile assistance for users. To this end, it is necessary to offer open and standardized connection protocols (USB, Bluetooth, Wi-Fi, among others) that allow stable and simple interoperability with external devices and provide the NP system with high compatibility. In addition, it is important to be able to establish bidirectional communication that allows the reception and sending of information in order to achieve effective coordination between devices. In the same aspect, physical interfaces are essential to ensure effective integration through a robust, secure and functional physical connection that allows interaction between devices. In this sense, the mechanical connection may require stable, secure, precise and aligned positioning to ensure correct interpretation of signals. This involves ensuring mechanical compatibility by considering shape, size and other physical aspects to ensure smooth ergonomic integration. Durability and resistance must also be considered, especially in clinical or rehabilitation environments where the technology is used intensively.
- Donning/doffing: donning and doffing time is one of the barriers limiting the use of NPs [44–46]. In fact, the time can range from a few minutes to about 1 h depending on the complexity of the setup [47]. This process encompasses everything from installing the system components to configuring it for immediate use. Typically, configuring the electrical stimulation parameters constitutes the most time-consuming aspect of a neuroprosthesis system. This has been addressed in the literature by developing automatic calibration algorithms using surface electrode arrays [44]. While literature reports on the matter are lacking, it’s commonly observed that doffing tends to be more expedient. Doffing typically entails simply removing the system, whereas donning requires a series of steps. These include precisely placing sensors, ensuring the comfortable positioning of various components of the NP system without impeding user movement, and prioritizing user comfort. Additionally, configuring the system for proper operation adds to the complexity of the donning process. Lack of information and guidance on the different NPs has also been identified as a limiting factor that can increase donning/doffing time [46]. This process is also a fundamental requirement in WRs [32, 48]. In this regard, donning time can be as long as about 30 min, while doffing usually takes about 10 min [32]. It should be noted that no evidence has been found on hybrid systems composed of NP and WR systems, but this may vary depending on the integration that has been designed between the two devices. However, it is essential to ensure that the joint assembly and configuration does not lead to a substantial increase in donning/doffing time in hybrid configurations to increase usability and user acceptance in the clinical environment.
- GUI: it is important to offer an intuitive and friendly interaction from a GUI, ensuring a more comfortable and effective experience for each user that employs it. In this sense, the use of visual or auditory feedback can help to know the status and operation of the system. The GUI should also allow the creation and development of sequences for specific activities and rehabilitation protocols. This can facilitate integration into daily activities and specific treatments. In addition, it must be possible to record and store usage data to improve performance and make future adjustments facilitating clinical evaluation and treatment, research and continuous improvement of the system. The system must be open and programmable allowing users or developers to program and modify the behavior of the system according to specific requirements. This encourages innovation and experimentation, allowing the implementation of tailored control strategies. In this sense, it should be possible to implement different control strategies both in open loop (predefined patterns) and closed loop (dynamic adaptation), as the choice of one mode or the other may depend on the clinical application and the user’s needs.
- Safety: safety is another key factor and it is a design priority to ensure the integrity of the user during use. The design should include robust features that protect the user from overstimulation, malfunction or potentially dangerous situations. It is important to pay attention to galvanic isolation, stimulation control mechanisms (intensity limits), emergency mechanisms (immediate stop of stimulation), protection and fault detection mechanisms (monitoring of system integrity and performance) and clear instructions for use. In the literature, it has been noted that systems employing implanted electrodes typically operate with a maximum pulse amplitude of 25 mA, whereas those utilizing transcutaneous electrodes operate within a range of up to 120 mA [1]. Pulse duration and frequency typically fall below 1000 µs and 100 Hz, respectively, as commonly observed [49–52].
- Battery autonomy: the system must be energy efficient in terms of consumption and have an adequate battery autonomy to ensure continuous and practical use by different individuals in their rehabilitation sessions. This implies that the different components of the system must have rechargeable batteries with a sufficient duration and it is desirable that they can be easily replaced. The required battery autonomy can vary depending on the specific application. However, existing literature frequently reports battery autonomy exceeding 4 h [8, 15, 21].
- Dimensions and weight: while there is no established standard or definitive studies defining acceptable size or weight criteria, literature suggests that portable electrical stimulators in modular systems typically have maximum dimensions of 356 cm^3^ and weigh less than 0.5 kg [8, 20, 21, 23–28] (Additional file 1: Table 1S). However, the specific requirements for these characteristics will vary based on individual needs and context of use. Given the aim of achieving a scalable system, it is essential to strive for the smallest possible dimensions and weight for each component to ensure wearability in all configurations. This enhances usability across stationary and mobile scenarios. In short, size and weight play pivotal roles in facilitating daily use, enhancing comfort and portability, and consequently, influencing user acceptability and mobility.

### Modular NP system suitable for hybrid FES-robot applications

#### Hardware

**Fig. 1:**
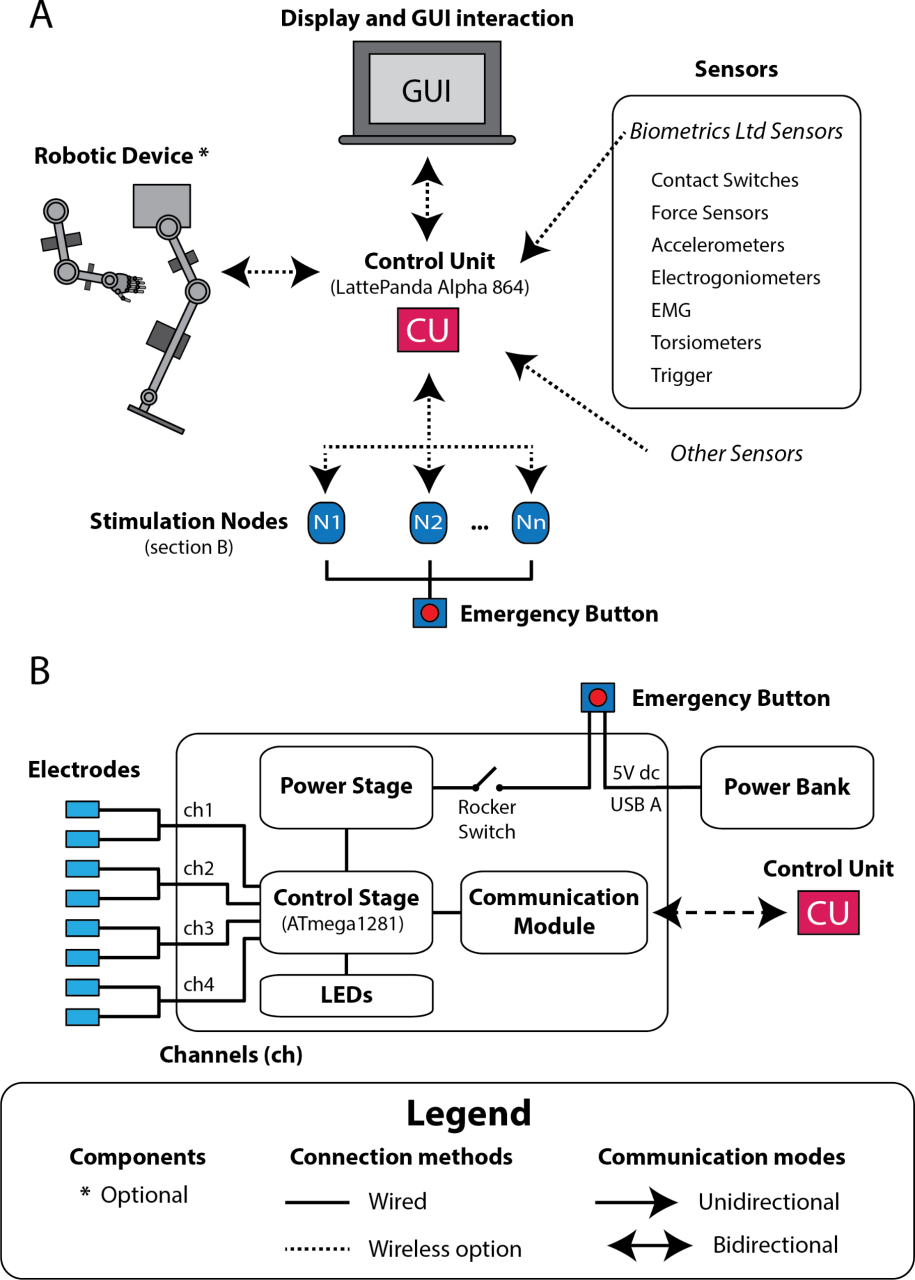
Modular NP system suitable for hybrid FES-robot applications. (**A**) System components, forms of connection offered and type of communication that can be established. The components marked as optional may not appear in some configurations of the modular NP system. (**B**) Main parts of the electrical stimulation node with their internal and external connections with an emergency button, multiple electrodes and a control unit (CU). Other abbreviations: graphical user interface (GUI), node (N), electromyographic (EMG)

The NP system consists of a modular architecture consisting of a sensor network, a CU where information is centralized and control strategies are implemented, and stimulation nodes directed to the use of transcutaneous electrodes (Fig. 1A and more technical information in section Technical specifications of our modular NP system of Additional file 1). For the sensor network, an external third-party system (Biometrics Ltd, Newport, UK [53]) has been integrated to allow the use of a wide variety of small portable wired or wireless sensors (Fig. 1A), although it is possible to integrate others in a simple way. ES nodes operate according to the performance requirements reported in the literature [54–56] and consist of an external battery, a connection for a common emergency stop button, an individual on/off switch, a power stage that feeds a control stage that modulates the ES into 4 galvanically isolated channels of electrical stimulation directed to transcutaneous electrodes, and a bidirectional communication module that connects to the CU (wired UART, Bluetooth or Wi-Fi), as shown in Fig. 1B. Everything is encapsulated by 3D printing for simple and fast assembly (Additional file 1: Fig. 1S A and B). The control unit (LattePanda Alpha 864 [57]) is a lightweight 3D encapsulated development board that can be powered through a power connection or an external battery and has a high capacity for the development and execution of the control software, as well as interconnection and communication features that facilitate the integration of other components and systems (Fig. 1A and Additional file 1: Fig. 2S).

#### GUI

The GUI runs on the CU and offers three modes of display and interaction: connecting it to a monitor with keyboard and mouse, via a direct touch screen, or remotely from a personal computer. These options accommodate various scenarios, ranging from situations where the control unit is not carried by the user to cases where it must be carried (Fig. 1A).

**Fig. 2:**
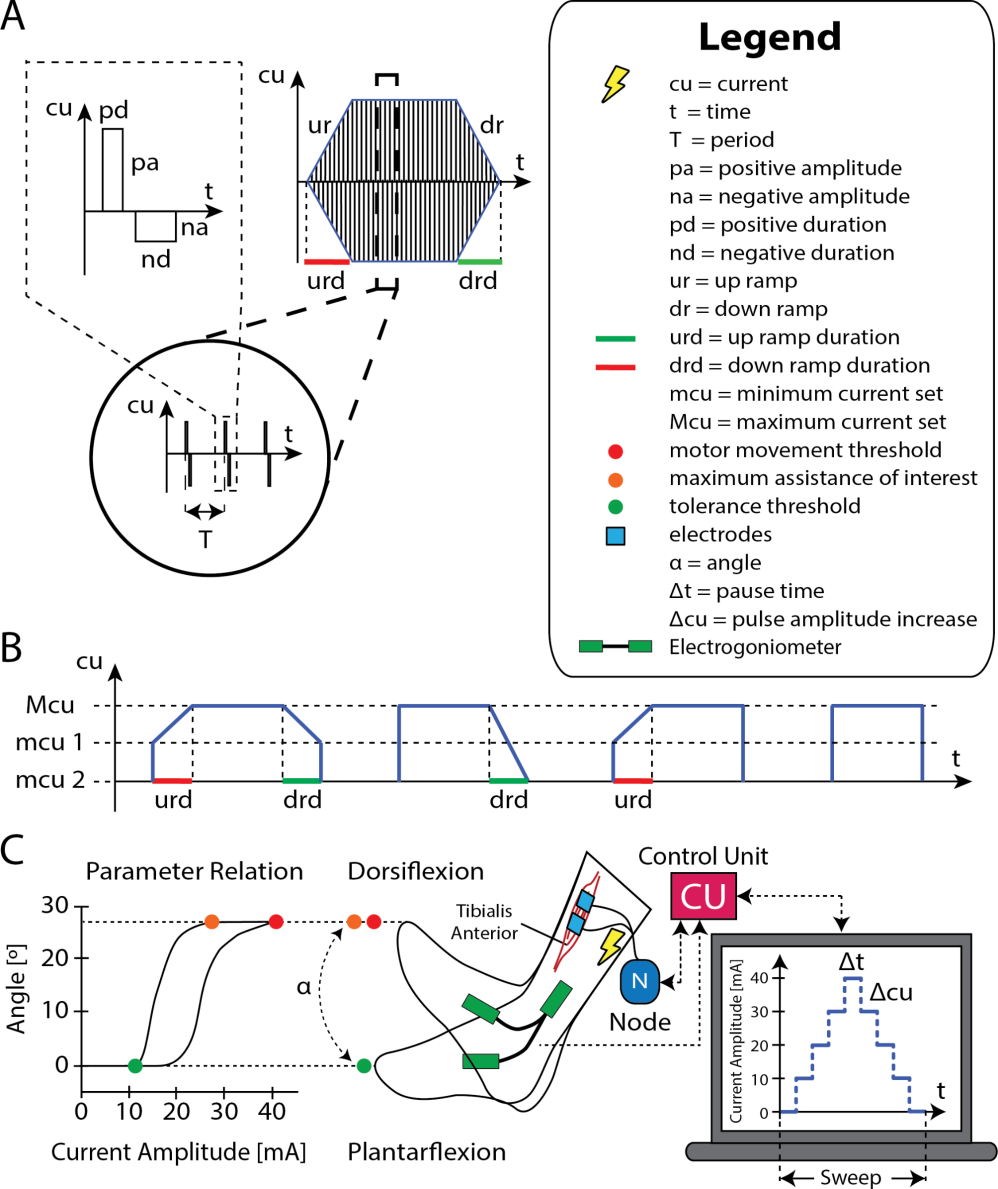
ES configuration: (**A**) fundamental parameters of the pulse trains that compose the ES, (**B**) envelopes of the types of pulse trains that can be configured and related parameters, and (**C**) example of the automatic pulse amplitude calibration method applied to the tibialis anterior

The GUI allows the configuration of the sensor network (types and quantity), defining the number of nodes together with the type of connection and communication (continuous, the received command is executed until another one is received, or punctual, the received command is executed only once) with the CU, the number of channels and relate each one to a muscle group, and select a control strategy from among those that have been implemented.

For ES, the GUI facilitates flexible configuration of the basic parameters for each channel (Fig. 2A and Additional file 1: Table 1S). It is possible to configure the pulse phase (monophasic, biphasic symmetrical or biphasic asymmetrical), amplitude, width, frequency (common for all channels or specific for each channel), repetitions per channel (single pulses, doublets and triplets [58, 59]), invert the pulse, or choose the order of stimulation for the node channels. Furthermore, it also allows defining more general characteristics of the pulse trains to generate tailored rectangular or trapezoidal profiles such as those shown in Fig. 2B. To this end, through the GUI it is possible to activate or deactivate up ramp and/or down ramp individually for each channel, to set the duration of each type of ramp independently and to define the minimum and maximum amplitude of ES for each channel (Fig. 2B).

The manual calibration of the ES parameters of each channel that is usually performed in the clinical setting, where a single parameter, usually pulse amplitude, is gradually increased until the maximum comfortable value specific to each muscle group for the user (comfort threshold) is reached, can also be performed from the GUI. However, this process is often subjective and may vary between therapists, so an automatic calibration method has been implemented in which the muscle response is characterized at a later stage (Fig. 2C). To do this, a position sensor or goniometer is selected in the GUI that relates to a particular muscle group, and the number of iterations required, the increment and the pause time with each increment are selected. With these parameters, a “sweep” is performed which consists of gradually increasing with the set increment from 0 until reaching the comfort threshold, previously calculated, making the established pauses with each increment, and then gradually returning in the same way to the 0 value of that ES parameter. This process is performed as many times as established in the number of iterations and, as a result, a relationship between muscle response and the selected parameter is obtained, which can be represented in real time (Fig. 2C). It has been observed that this relationship presents hysteresis and that there is a maximum of effective assistance, beyond which the muscle response (position reached) does not increase (Fig. 2C) [60]. In general, it is below the comfort threshold, so its use could delay the onset of muscle fatigue associated with ES compared to the use of the comfort threshold itself.

The GUI facilitates joint range of motion tests using sensors. Range of motion tests include passive (recording maximum joint movement), activity-associated (measuring range of motion during specific activities), and FES-assisted (automatically recording range of motion during assistance calibration). It also enables saving and loading of settings, facilitating quick configuration of the modular NP system for different users in future sessions, and allows viewing, recording, and reviewing system usage data. When a stimulation node network is configured, the system logs information from sensors and robotic devices, including pacing data during activities. The GUI includes an information tab for inexperienced users, providing insights into functionalities and ES settings. It incorporates error detection and visualization strategies, issuing informative messages and saving them for optimization. Safety measures, such as defined value ranges and manual calibration for ES pulse amplitude, prevent invalid data entry. Adjustable ramp amplitudes are constrained below the comfort threshold, triggering warnings for inappropriate values.

### Modular and hybrid NP applied to gait

This section presents in detail the adaptations implemented in the previously presented system for use during gait in combination or not with a lower limb WR.

#### Modular NP applied to gait

**Fig. 3:**
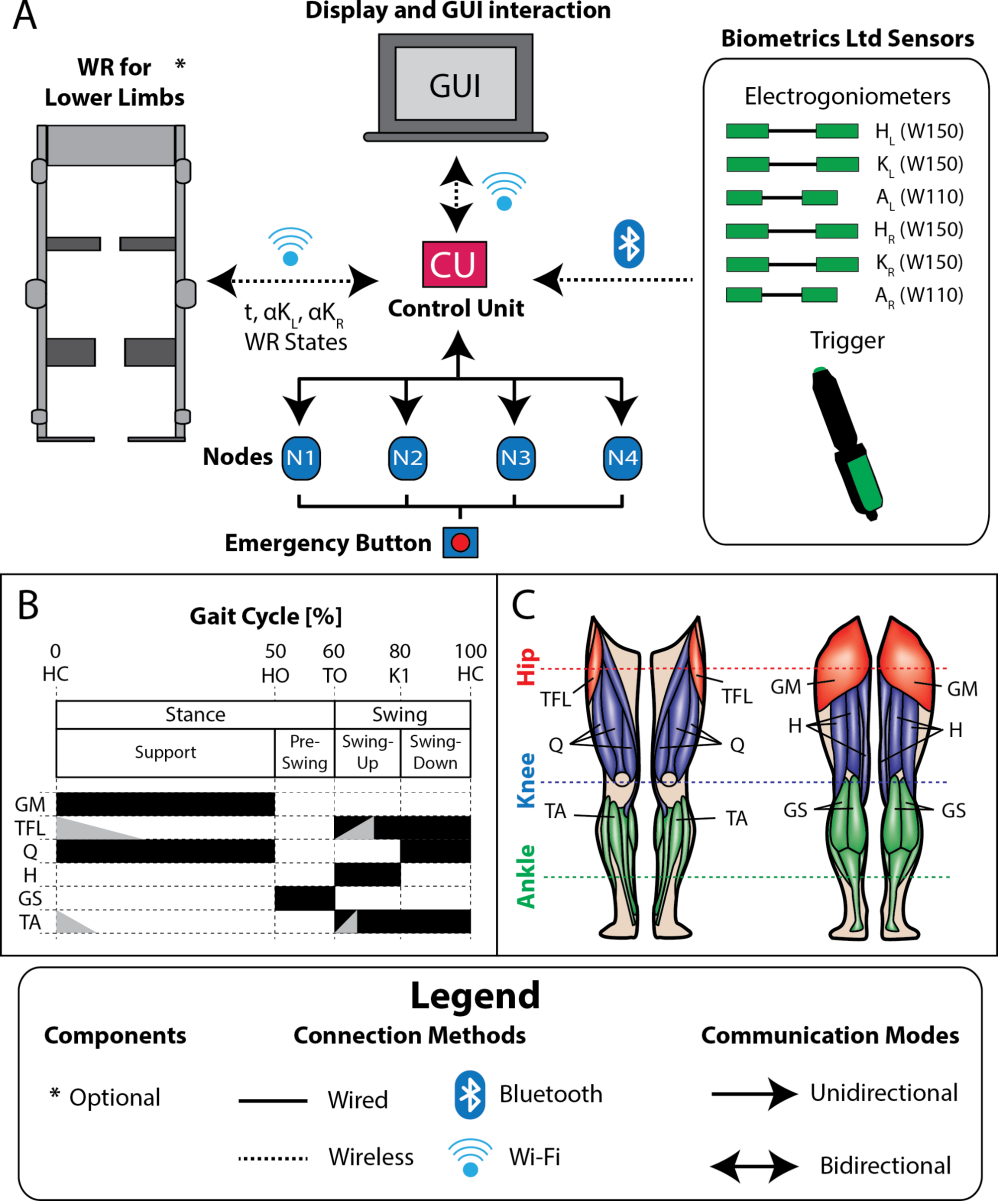
Modular NP system adapted for gait activity. (**A**) System components, how they are connected and the type of communication that is established. (**B**) Events and gait phases identified with the preset event detection algorithm and finite state machine used in open-loop control to assist the target muscle [61, 62]. The predefined assistance is marked in black. The example of two up and down ramps down is shown in grey. The down ramps are executed once the end of stimulation is detected. The up ramps, on the other hand, would be executed within the black band as the corresponding event is detected. (**C**) Target muscle groups of the lower limbs involved in gait. Abbreviations: heel contact (HC), toe off (TO), heel off (HO), maximum knee flexion in swing (K1), gluteus maximus (GM), tensor fasciae latae (TFL), quadriceps (Q), hamstrings (H), gastrocnemius (GS) and tibialis anterior (TA), time (t), left knee angle (αK_L_), right knee angle (αK_R_), left hip (H_L_), left knee (K_L_), left ankle (A_L_), right hip (H_R_), right knee (K_R_) and right ankle (A_R_)

For the specified application, a sensor network comprising up to 6 wireless electrogoniometers (W110 for ankles and W150 for hips and knees, Biometrics Ltd, Newport, UK [53]) was configured to measure the angular motion of each lower limb joint at a frequency of 100 Hz during gait (Fig. 3A). Consequently, three electrogoniometers, one for each joint, needed to be positioned on each leg (Fig. 3A). The sensors, easily affixed to joints with hypoallergenic double-sided adhesive tape, operate effectively within a 30 m range to the USB receiver. Validated for measuring lower limb joint angles during walking, their angular data proves valuable for developing closed-loop control algorithms in this context [63–67]. As for the electrostimulators network, a configuration of up to 4 nodes was established as sufficient for this context, considering the number of channels per node and the main muscle groups involved in the gait activity. Because of this, a certified emergency button (XW1E-BV404M-R [68] from IDEC) was chosen (Fig. 3A) and encapsulated by 3D printing (Additional file 1: Fig. 1S B and Fig. 1S C).

Considering the need to act in the different phases and sub-phases of gait, it is important to note that these sub-phases can represent up to 10% of the gait cycle [69]. The average gait cycle duration in adults is between 0.98 and 1.07 s [70]. This therefore translates into time limits of around 100 ms. Characterization tests were performed for each of the connection methods between the CU and the pacing nodes to determine which was optimal in this context.

Some additional functionalities and algorithms of interest for this context were implemented in the GUI. A gait event detection algorithm was integrated to segment the gait in real time and to be able to use this information for automatic assistance management by the NP or hybrid system. This algorithm had a one-sided heuristic event detection approach using the angular trajectory information of the hip, knee and ankle and the joint range of motion obtained from the range of motion test during activity. Accordingly, two predefined modes of sensor network configurations were enabled. The first one, consisting of three electrogoniometers to be located at the joints of one of the lower limbs, and the second one, consisting of six electrogoniometers to be located at the joints of both lower limbs. The unilateral approach was intended to reduce the number of sensors, reducing donning/doffing time and providing more comfort to the individual in cases where the need for unilateral assistance was identified only. This algorithm relied on adaptive thresholds to segment the gait into support, pre-swing, swing-up and swing-down sub-phases (Fig. 3B). To achieve this segmentation, the algorithm detects four events shown in Fig. 3B: heel contact, heel off, toe off and maximum knee flexion during swing.

The muscle groups targeted for assistance during gait were selected due to their relevance in this activity and in the joint movement of the lower limbs (hips, knees and ankles) and considering the feasibility to assist them using superficial electrodes. The targeted muscle groups included the gluteus maximus, tensor fasciae latae, quadriceps, hamstrings, gastrocnemius and tibialis anterior muscles, as shown in Fig. 3B and C. These muscle groups could be related to the configured channels of the node network, as discussed above. This was done in order to implement a preset one-sided finite state machine type open-loop control algorithm (Fig. 3B). This algorithm relates each muscle group to a specific way of stimulation depending on the gait sub-phase. The algorithm employed information from the event detection algorithm, the network configuration of nodes and channels, and the relationship between the identified muscle groups and the selected channels to manage the ES assistance.

The finite state machine was based on the research of Kesar et al. and Bogataj et al. [61, 62] with added modifications of clinical interest. It is named as the standard mode (Fig. 3B) and consists of a predefined state machine that ipsilaterally assists the musculature based on angular information from the ipsilateral hip, knee and ankle joints. Here it can be seen that gluteus maximus and quadriceps are assisted during the stance phase to maintain posture and help the lower limb not to flex from hip and knee extension, respectively. Tensor fasciae latae assistance was predefined during the swing with the aim of helping the leg to move forward by producing hip flexion, although this choice was not based on information found in the literature. In fact, it is a deep muscle group that is complicated to assist superficially. Hamstrings assistance was predefined in the swing-up to assist in knee flexion. The gastrocnemius muscles are assisted in the toe-off phase to increase plantarflexion and improve propulsion, also helping to improve leg drive in the swing. And the tibialis anterior is assisted in the swing to aid ankle dorsiflexion and achieve greater ground clearance to prevent foot dragging or stumbling. The control algorithm integrates a variant of the standard state machine that we call cross mode, which consists of assisting the ipsilateral leg using the gait events of the contralateral side. The control algorithm integrates a variant of the standard state machine that we call cross mode, which consists of assisting the ipsilateral leg using the gait events of the contralateral side. This mode needs the angular information of all the joints of both lower limbs and for this study was only adapted for gastrocnemius, where we wanted to test the assistance with a time advance to achieve a better plantarflexion, due to the reduced duration of the pre-swing sub-phase. For this purpose, contralateral heel contact was used to start the assistance and was stopped with ipsilateral toe-off.

#### WR combination

The modular NP was combined with a knee-powered exoskeleton prototype developed by ABLE Human Motion, S.L. It consists of motorized knees and articulated hips and ankles that allow movement in the sagittal plane [71–73]. This lightweight (9.8 kg) WR is easy to don (4 min 43 s) and doff (2 min 7 s), and is designed to provide assistance during standing, walking and sitting tasks [73]. The therapist can initiate steps in gait assistance mode manually via buttons on the lumbar segment of the WR, from its integrated ABLE Care mobile app, or automatically by detecting the user’s intention [71]. Furthermore, within the application, users can configure various control parameters of the WR, including the sensitivity of step intention detection, the maximum knee flexion angle during swing, which adjusts the predefined trajectory for the WR during the swing phase, and the swing execution speed. Throughout the gait cycle, the WR maintains the leg fully extended during the stance and offers flexion-extension assistance during the swing phase by adhering to the predefined trajectory of the knee joint angle [73, 74].

The knee-powered exoskeleton prototype only offered a wireless local area network connection via the IEEE 802.11 standard, which conflicted with our Wi-Fi. Therefore, we opted for a wired UART connection between the CU and the ES nodes in the hybrid configuration in order to be able to use WLAN bidirectional communication with the WR. This communication could be periodic (measurements are received periodically without the need to request them) or in a question-answer mode (these measurements must be requested from the WR). Through this communication network, the modular NP received real-time data at a frequency of approximately 100 Hz regarding the angular position of the knees and the status of the WR (stance, left swing and right swing), as illustrated in Fig. 3A. This information, together with the angular information from the hips and ankles, was used to develop standard hybrid mode and cross-hybrid mode control strategies, related to the control strategies previously explained in Sect. 2.3.1. In these different variations, the WR determined the stance and swing phases, while the gait event detection algorithm implemented in the NP identified the subphases. Thus, during the hybrid system’s use, the WR identified the intention to step and initiated the step by assisting during the swing phase. Additionally, the WR communicated to the NP the current gait phase of each lower limb, and the NP identified the subphases and assisted based on the predefined state machine described in Sect. 2.3.1. This approach achieved a coordinated hybrid system in which the NP system adapted to the WR’s operation.

Since the WR did not provide angular information at hip and ankle level, it was necessary to set up two additional predefined sensor network configuration that could be loaded quickly. The first, consisting of two electrogoniometers to be located at the hip and ankle of one of the lower limbs, and the second, consisting of four electrogoniometers to be located at the hips and ankles of both lower limbs. Additionally, electrogoniometers needed to be ensembled on these joints. Custom 3D printed parts were developed for the standardized physical integration (Additional file 1: Fig. 3S to 9 S) without affecting the fit and function of the WR. All these parts were designed with the aim of facilitating the combination of the systems in a simple, fast, stable and safe way.

### Technological personalization process

**Fig. 4:**
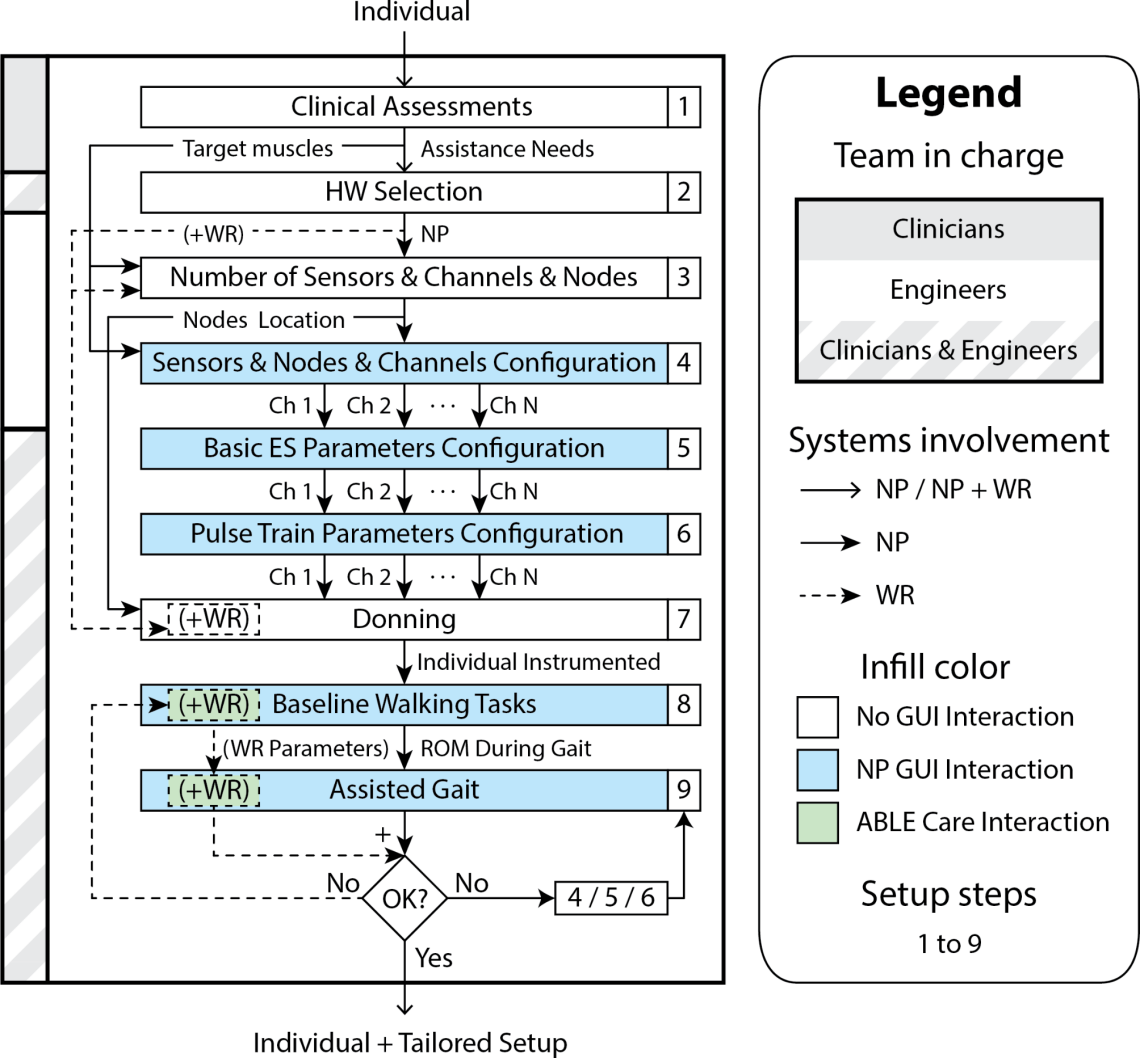
Personalization process to identify the technology of interest to the assistance and configure it appropriately. The diagram indicates the team involved in each of the tasks detailed, the configuration steps (1 to 9) applied to each of the systems and whether it is necessary to interact with the system through the GUI. In the case of the knee-powered exoskeleton prototype GUI, this is ABLE Care, as discussed in Sect. 2.3.2. Abbreviations: hardware (HW), neuroprosthesis (NP), wearable robot (WR), channel (Ch), range of motion (ROM) and graphical user interface (GUI)

The personalization process (Fig. 4) relies on experience and clinical assessments to identify and configure the technology of interest for each individual’s gait assistance (modular or hybrid NP). The steps are described below, the first two of which are common to both options.

Step 1 (Fig. 4 “Clinical Assessments”). The individual is assessed by the clinical team to determine the level of disability, abilities and needs. The outcome of the evaluations performed by the clinical team detailed by Herrera-Valenzuela et al. in [75], help to identify the most affected musculature whose contribution to gait is altered. This information, together with knowledge about the different control loops offered by the modular NP system, helps to identify the target muscle groups for FES assistance.

Step 2 (Fig. 4 “HW Selection”). Based on the identified needs, the clinical team and the engineering team collaboratively evaluate the hardware options (modular or hybrid NP) of interest to use during gait activity. Several options may be considered for the same individual.

Step 3 (Fig. 4 “Number of Sensors & Channels & Nodes”), the number of sensors and the number of nodes and stimulation channels required to assist the target muscle groups identified in Step 1 are determined. First, the number of sensors is determined. Recall that the event detection algorithm and control algorithms used in the gait-adaptive NP system have a one-sided approach (Sect. 2.3.1 and 2.3.2). This is important as a preset sensor network of 3 to 6 electrogoniometers can be selected for gait assistance with the modular NP system, or 2 to 4 goniometers for the hybrid system (Sect. 2.3.1 and 2.3.2). The selection of one or the other configuration therefore depends on the needs identified in Step 1 and the hardware selection made in Step 2 of this process. Second, the number of channels required is determined, which should match the number of target muscle groups identified in Step 1. In collaboration with the clinical team, different possibilities are established, i.e., different combinations of muscles and channels to vary the configuration of the system in case the assistance is not optimal for the individual and needs to be adjusted during activity. Thirdly, depending on the location of the muscle groups to be assisted and the number of channels required, the number of nodes required and their most appropriate placement is determined. For example, for an individual who needs bilateral assistance in tibialis anterior and gastrocnemius of both legs, 4 channels are needed for which one node placed in the lumbar area can be used, minimizing the weight of the system, or two lateral nodes (two channels per node), reducing the length of the wiring and improving comfort during the activity. The aim is to optimize and balance the weight that the individual will subsequently bear when walking with the defined system, as well as the time required for donning/doffing.

Step 4 (Fig. 4 “Sensors & Nodes & Channels Configuration”). The engineering team configures the sensor network and the network of nodes and channels through the GUI based on what was determined in the previous step. The preset sensor network of sensors chosen in Step 3 is loaded. In relation to the stimulation nodes, the connection method and the communication mode are defined for each of them. Each channel is also associated with one of the target muscle groups identified in Step 1.

Step 5 (Fig. 4 “Basic ES Parameters Configuration”). The clinical team places the surface electrodes on the target muscles and both teams will collaborate in setting the ES parameters through the GUI (Fig. 2A and C). The parameter setup consists of setting the pulse phase for each node, defining a common and/or independent frequency per channel, setting a common pulse width for all channels per node, and performing pulse amplitude calibration.

Step 6 (Fig. 4 “Pulse Train Parameters Configuration”). Once these basic ES parameters have been determined, it is determined when and on which channels up ramps or down ramps are to be used and the parameters relating to them are adjusted (Sect. 2.2.2 and Fig. 2B). For this, it is necessary to consider that the ES is controlled with an open loop based on the default finite state machine (Fig. 3B).

**Fig. 5:**
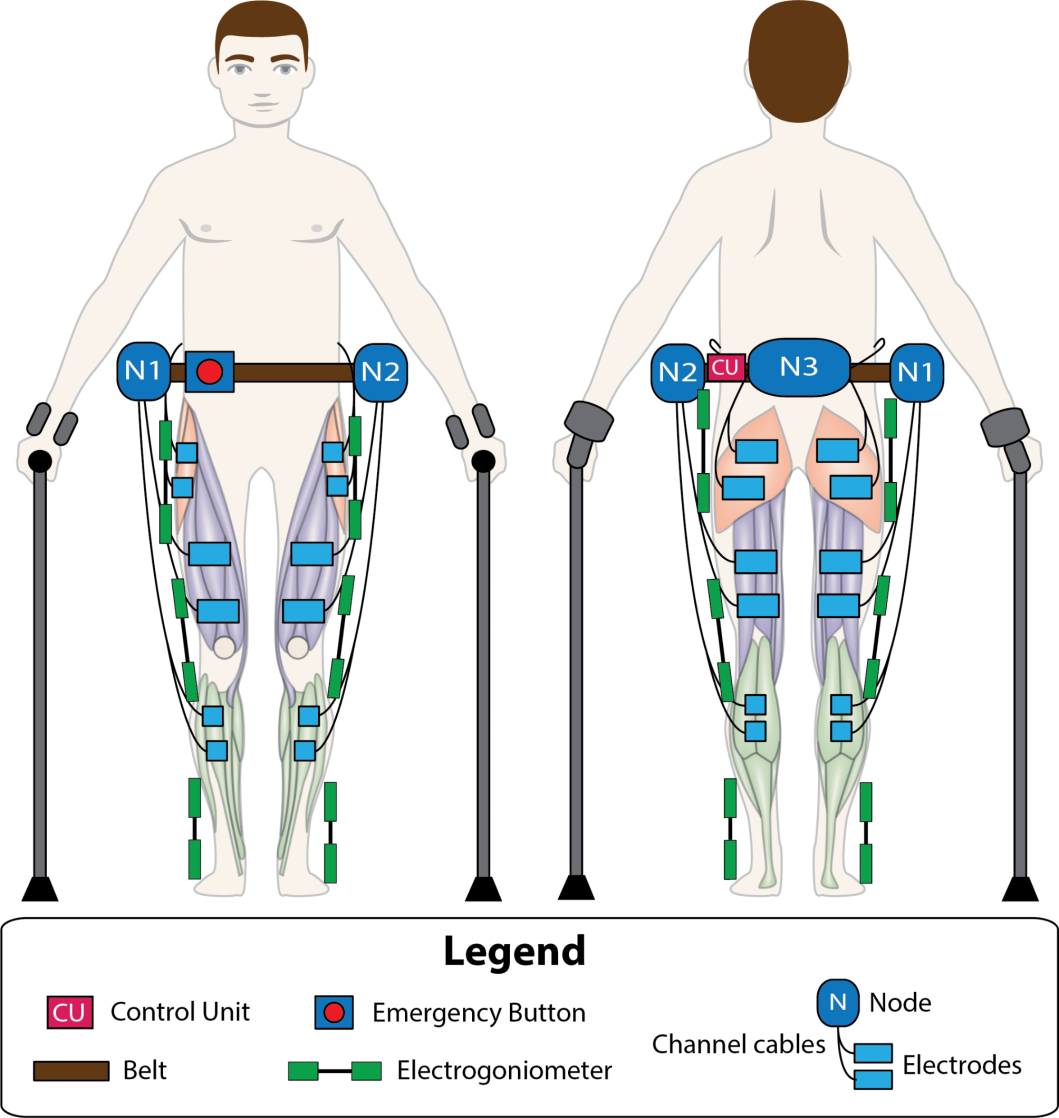
Example of an individual wearing a belt with the modular NP system adapted for gait with the following configuration: 6 electrogoniometers, 3 nodes, the CU, the emergency button and 12 channels. The wiring from the emergency button to the nodes and from the nodes to the CU have been omitted for simplicity of the figure

Step 7 (Fig. 4 “Donning”). Both teams collaborate to mount the modular or hybrid NP system on the subject. If the chosen system is the modular NP system, both teams place the sensor network over each joint and the CU, the nodes and the emergency button on a belt with the 3D parts designed for that purpose (Additional file 1: Fig. 1S B.1). Figure 5 shows an example of donning of an individual with a possible hardware configuration of the modular NP system in which the entire target musculature of the lower limbs needs to be assisted. In case a hybrid configuration has been chosen, the modular NP system needs to be placed over the WR with the 3D parts specifically designed for this purpose. For the knee-powered exoskeleton prototype used in this hybrid form, it will be necessary to use the 3D parts detailed in the supplementary material (Additional file 1: Fig. 1S to Fig. 9S). However, many of the parts can be pre-placed as they do not affect the WR donning and operation, saving donning time when hybrid assistance is required. The parts that can remain in place on the WR prior to donning process after the first installation are detailed in the corresponding figures (Additional file 1: Fig. 4S to Fig. 9S). With these parts in place, the WR is first positioned, adapting the dimensions to the anthropometry of the individual. Next, the electrogoniometers are placed on the hip and ankle unilaterally or bilaterally with the remaining pieces designed for this purpose (Additional file 1: Fig. 4S to Fig. 7S). Finally, the nodes (Additional file 1: Fig. 1S) are placed in the central assembly part (Additional file 1: Fig. 8S) and/or lateral assembly part (Additional file 1: Fig. 9S), as intended, and the CU (Additional file 1: Fig. 2S) is placed in the central assembly part (Additional file 1: Fig. 8S).

Step 8 (Fig. 4 " Baseline Walking Tasks “). Both teams calibrate the modular NP system or the hybrid configuration. In the case of the modular NP, the system is calibrated to ensure correct detection of gait events. To this aim, the electrogoniometers are calibrated in an upright static position and the sagittal range of motion of the subject’s lower limb joints is recorded during unassisted walking. In case a hybrid configuration is used, the electrogoniometers are also calibrated in static upright position with the WR on and the sagittal range of motion of the subject’s lower limb joints is recorded during walking with the WR. In addition, these steps are used to adjust the WR operating parameters (Sect. 2.3.2) from ABLE Care during gait to achieve a more natural and fluid motion.

Step 9 (Fig. 4 “Assisted Gait”). Both teams collaborated in the choice of the control strategy to be used, depending on the configuration applied (modular or hybrid NP). For the first case, a choice was made between standard or cross mode (Sect. 2.3.1), while for the second, a choice was made between standard or cross hybrid mode (Sect. 2.3.2).

With the support of both teams, the individual walked with each of the set configurations. However, it was possible to make updates and adjustments to the operation of the system based on observations made, clinical judgment, and feedback from the individual. Once the system tuning was completed, a personalized configuration was obtained based on the individual’s needs. In this way, different personalized configurations can be designed in the first session that can be saved and quickly loaded in subsequent sessions. Thus, from the first session, it is only necessary to load the saved personalized configuration, calibrate the pulse amplitude for each muscle group (Step 5), put on the system (Step 7) and perform the gait activity (Step 9).

### Experimental testing and analysis

This section presents the evaluation of the proposed modular NP system by comparing it with other similar systems found in the literature.

In contrast, to assess and verify the functionality and adaptability of our system, whether used in conjunction with a robotic device or as a standalone solution, we carried out a series of experiments involving individuals with diverse functional requirements. These experiments are detailed in the next subsection.

#### Experimental validation procedure in gait activity

For the experimental validation, the technological personalization process was followed to identify the configurations of interest for gait assistance for individuals with SCI and stroke (Table 1). This study had four participants: two individuals with SCI were recruited at the National Hospital for Paraplegics (Toledo, Spain) and two individuals with stroke were recruited at the Institut Guttman (Badalona, Spain). The selection of these two distinct patient populations with diverse assistance requirements is rationalized by the pursuit of a technology capable of adapting and personalizing to each patient’s individual needs. Consequently, both patient groups were incorporated into the experiments to assess the system’s versatility and personalization capacity across various clinical scenarios and a spectrum of functional needs. Table 1 summarizes the information about each individual and the configuration adopted for each of them.

**Table 1:** Participants information. Individual (I), spinal cord injury (SCI), gender (G), female (F), male (M), weight (W), height (he), walking index for spinal cord Injury (WISCII), functional ambulation categories (FAC), channel (Ch), muscular group (MG), comfort threshold (CT), up ramp duration (urd), down ramp duration (drd), tibialis anterior (TA), gastrocnemius (GS), hamstrings (H), wearable robot (WR)

							Modular NP configuration		WR - knee-powered exoskeleton prototype
I	G	Age	W [kg]	H [cm]	ASIA AIS	Clinical assessments	Nodes	Ch - MG - CT [mA] - urd [ms] - drd [ms]	
SCI 1	F	67	69.4	150	D	WISCII − 19	1	**Node 1** Ch1 - TA − 55–100–100 Ch2 - GS − 45–50 -50 Ch3 - H − 65–100–100	-
SCI 2	M	44	80	174	D	WISCII − 20	2	**Node 1** Ch1 - TA − 28–100–100 Ch2 - GS − 40–50 -50 **Node 2** Ch1 - TA − 23–100–100 Ch2 - GS − 39–50 -50	-
Stroke 1	M	43	64.5	174	-	FAC − 4	1	**Node 1** Ch1 - TA − 30 − 0–0 Ch2 - GS − 40 − 0–0 Ch3 - H − 40 − 0–0	65° Maximum knee swing flexion Flexion-extension ratio 50/50
Stroke 2	M	59	83	180	-	FAC − 4	2	**Node 1 & Node 2** Ch1 - TA − 60 − 0–0 Ch2 - GS − 61 − 0–0 Ch3 - H − 77 − 0–0	

The experimental protocol (CEIC-CHTO, no. 716 26/05/2021) was approved by the by the Institutional Review Board at the Ethics Committee of the Hospital Complex of Toledo, Spain. All participants signed an informed consent before the start of the experimental protocol, which comprised one or two sessions, with clinical assessments being the first task in both cases. The number of sessions varied according to the abilities and needs identified during the clinical assessments. For individuals who exhibited a high degree of difficulty in gait activity and greater assistance needs, the clinical team determined the need for WR use and participation in two sessions. The first was used to familiarize (training session) with the use of the WR and optimize the anatomical fit and control parameters of the WR (Sect. 2.3.2) to achieve a comfortable and smooth automatic gait coordinated by movement intention detection. The second session took place at the most one week later and addressed the recording of unassisted and assisted gait. In this second session, the technological personalization process associated with the modular and hybrid NP system was applied. In addition, system usage data (Sect. 2.3.1) were recorded for at least three 10-meter walking corridors with each of the employed configurations. For individuals able to walk with minimal assistance, the clinical team determined that the optimal assistive technology was the modular NP and performed a single session. This was the case for individuals with SCI able to walk with minimal assistance (WISCI II > 19). In this session, the aforementioned technological personalization process for the modular NP system was applied and system usage data (Sect. 2.3.1) were recorded for at least three 10-meter walking corridors with each of the employed configurations. All gait tests were performed at the speed selected by the user and with as few assistive devices and orthoses as possible, always under the supervision of a trained clinician. Where necessary, the clinician was able to assist the user in maintaining balance by holding the user by the lumbar section of the WR.

Regarding the configuration of the modular NP system, the choice of the number of sensors was not relevant for this study, since the objective was to record the complete movement during walking for later analysis, so 6 electrogoniometers were used in a generalized way. Once the number of channels and nodes and the physical location of the nodes were determined, they were configured following the personalization procedure (Sect. 2.4). A wired connection and continuous communication mode between nodes and the CU were defined. The use of symmetrical biphasic pulses was established as the safest and most recommended [10]. Next, a stimulation frequency of 40 Hz and a pulse width of 250 µs common to all channels of each node were selected. Then, the clinician placed 5 × 5 cm self-adhesive surface electrodes on the muscle groups of interest and manual ES pulse amplitude calibration was performed (Sect. 2.2.2). With this, the comfort threshold in each channel was identified for each individual and set as the upper pulse amplitude limit (Table 1). Where ramps were used, the lower pulse amplitude limit was set to 0 (Table 1). The system was then donned, calibrated and different optimal configurations of interest were defined by iterative adjustment of the assistance based on clinical judgment, observation of the gait pattern and user feedback.

#### Experimental analysis

The effect on gait at the kinematic level was analyzed and the results obtained were compared with the clinical objective pursued in proposing this configuration. The records of each participant were processed with MATLAB R2021b, segmented into gait cycles, and the kinematic characteristics such as maximum ankle dorsiflexion at heel contact, plantarflexion angle at toe-off, maximum dorsiflexion during mid-swing and maximum knee flexion during swing, were obtained [76, 77].

These kinematic characteristics were analyzed with SPSS Statistics 26. First, normality (Shapiro Wilks test) and sphericity (Mauchly test) tests were performed. Subsequently, the variables were compared between the different configurations used for each individual. Descriptive statistics such as mean (M) and standard deviation (SD) were calculated. In all cases there were more than two conditions to compare, so a repeated measures ANOVA was applied. In cases where the sphericity criterion was not met, a Huynh-Feldt correction was applied when the Greenhouse-Geisser test showed an epsilon greater than 0.75. Otherwise, a Greenhouse-Geisser correction was applied. To identify between which conditions significant changes occurred, a post hoc pairwise comparison analysis with a Bonferroni correction was applied. In cases where the normality criterion was not met, the Friedman test for nonparametric samples was applied. In this case, to identify conditions in which significant differences occurred, post hoc tests were performed with the Wilcoxon test.

## Results

### Technical results of the modular NP system

The development of this new NP system has resulted in a modular architecture system whose complete technical characteristics can be reviewed in Table 1S of the Additional file 1, where it compares with the other systems of similar approach found in the literature [8, 15, 21–26].

This system has been able to integrate a third-party sensing system that allows communicating a great variety and quantity of light (< 100 g) sensors through wired or wireless connection. In addition, thanks to the CU’s own characteristics, it is possible to connect other sensors in a simple way or other third-party systems, as has been the case of the integration of the sensors carried by the knee-powered exoskeleton prototype itself.

Four-channel stimulation units have been developed using transcutaneous electrodes that meet the performance requirements in the context of FES applications reported in the literature. These nodes allow wired or wireless connection (Bluetooth or Wi-Fi IEEE 802.15.1) to establish bidirectional communication with the CU. They are equipped with a fast-charging external battery (3 h) that has been experimentally proven to provide each node with a battery autonomy of about 33 h under continuous electrical stimulation conditions at maximum demand (3.6 W). Moreover, being interchangeable, continuous use is favored by the availability of spare batteries. These nodes have been encapsulated by 3D printing with a design that allows easy access to the electronics for repairs or hardware upgrades, quick change of the external battery and simplifies safe placement. The final dimensions of the nodes have been 149 × 85 × 89 mm. The weight without battery was 255 g and the weight of the selected battery 225 g.

The CU used meets the fundamental characteristics for the flexible and scalable integration of the two components mentioned above due to its high connection capacity and the communication protocols it offers. In addition, characterization tests for each of the connection methods between the CU and the stimulation nodes revealed that between the arrival of information from the sensors and the application of the ES, delays of approximately 11 ms, 60 ms and 25 ms were obtained for wired UART, Bluetooth and Wi-Fi connection, respectively. Therefore, the wired or Wi-Fi connection was the most interesting for applications where quick action is required, such as walking. In addition, it allows its combination with robotic devices, having achieved a successful integration with a knee-powered exoskeleton prototype developed by ABLE Human Motion. Thanks to its capabilities, it has enabled the implementation of algorithms for data capture and processing (gait event detection algorithm), as well as the implementation of complex operations and integration of control algorithms (standard, cross, standard hybrid and cross-hybrid open loops). In addition, it has allowed the development of an intuitive GUI that facilitates user interaction with the system. The CU has been encapsulated by 3D printing, which has also enabled it to be powered by a fast-charging external battery (2.4 h) that gives it a battery autonomy of 24 h. However, it is possible to connect the CU to a power outlet in cases where the user does not need to carry such a system. The final dimensions of the control unit with the 3D package were 234.4 × 125.5 × 66 mm. The weight without battery was 378 g and the weight with battery was 248 g. The weight was comfortable and the dimensions were adequate for use in the gait application, as referred by some users [75].

The 3D printed design has facilitated donning/doffing process of the system in several modalities. Firstly, independently on a belt that fits the person. Secondly, it has allowed easy, efficient, stable and safe assembly of the modular NP system on a WR (knee-powered exoskeleton prototype developed by ABLE Human Motion) that supported it without negatively impacting the donning or operation of the WR. The hybrid assembly time can be consulted in Table 2, which considers that a single person has to assemble the NP system on the WR and has to place an electrogoniometer on each hip and ankle, three stimulation nodes and the CU. In relation to the whole donning/doffing process, which includes the complete configuration of the system in its stand-alone or hybrid form, the times were documented and can be consulted in [75].

**Table 2:** Hybrid assembly time. This table shows the times required to assemble the systems using the 3D parts designed for this purpose (additional file 1: tables 4 S to 9 S)

System assembly phase	Measured times [s]
Donning/doffing of a hip goniometer over WR	30 / 30
Donning/doffing of an ankle goniometer over WR	30 / 20
Donning/doffing of an ES node over 3D printed part	5 / 5
Donning/doffing of CU over 3D printed part	5 / 5
Whole assembly donning/doffing	140 / 120

An intuitive GUI has been provided to allow system configuration (connections, communications and components) in an efficient and fast way by loading preset ES settings or protocols that can help streamline therapy sessions. In addition, apart from the basic ES configuration functionalities, it allows manual and automated calibrations that can assist in the objective and accurate tuning of the ES. Relevant information has also been included to guide the user in the use of the system and it has been possible to implement different range of motion tests of clinical interest. In addition, the system was able to satisfactorily record usage information, which has been used for further analysis in a gait application.

Finally, the necessary physical and software resources have been made available to guarantee the safety of the system’s use. At the physical level, an emergency button has been enabled to be connected to each ES node, which allows to immediately stop the assistance in a common way in case of any risk or need situation, although it was not necessary to use it during the validation tests. Thanks to this connection, a 4-way emergency button, encapsulated in 3D for easy assembly and with a final weight of 180 g, has been specifically tested for 4 ES nodes in the running application. At the software level, safe limits, not allowed characters and parameters and calibration conditions have been established to ensure the proper operation of the system.

### Validation results in gait application

**Fig. 6:**
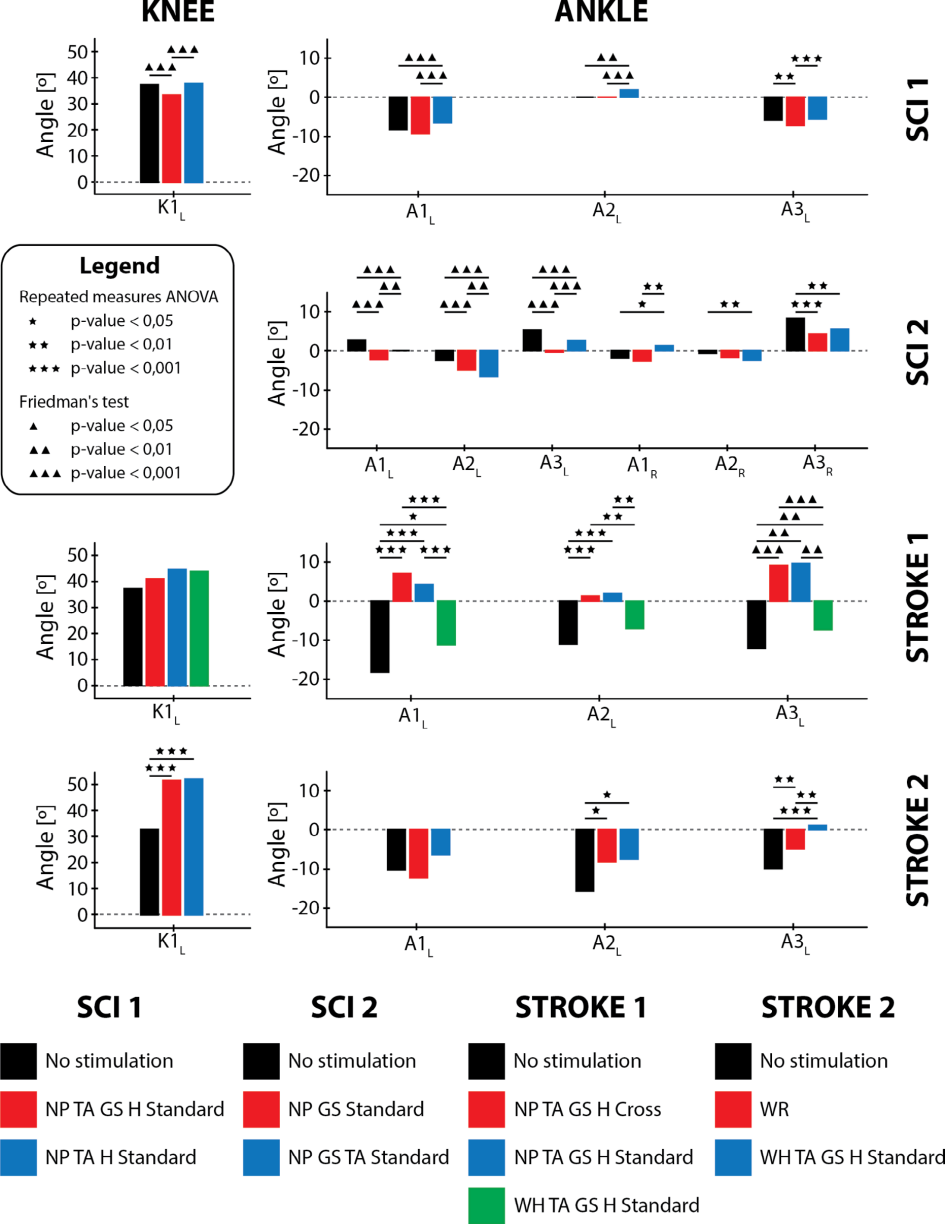
Results of the kinematic impact observed with the application of the different configurations tested for each individual and their statistical significance according to the statistical test used. Knee flexion and ankle dorsiflexion correspond to positive angle values, whereas knee extension and ankle plantarflexion correspond to negative angles. Thus, the maximum dorsiflexion will be represented by positive values, while maximum plantarflexion will be indicated by negative values. Abbreviations: maximum knee flexion in swing (K1), ankle angle at heel contact (A1), ankle angle at toe off (A2), maximum dorsiflexion in mid-swing (A3), left (L) and right (R), neuroprosthesis (NP), wearable robot (WR), wearable hybrid (WH), hamstrings (H), gastrocnemius (GS) and tibialis anterior (TA)

These tests were performed with the purpose of demonstrating the suitability of the system and the usefulness of the technological personalization process employed to facilitate the configuration of the system to the user’s needs. We do not intend to make an in-depth analysis or inferences about the potential benefits at the clinical level that are already addressed in the study proposed by Herrera-Valenzuela et al. [75]. However, we briefly present four cases and a small analysis showing the results of the application of different configurations obtained through the technological personalization process and their kinematic impact on the gait pattern (Fig. 6).

Specific configurations of assistance were obtained for each individual based on the technological personalization process. The different configurations aimed to improve mainly maximum ankle dorsiflexion at heel contact, plantarflexion angle at toe-off, maximum dorsiflexion during mid-swing, and maximum knee flexion during swing. Statistical results supporting the comments detailed below in this section and highlighted in Fig. 6 can be found in the supplementary material (Additional file 1: Tables 2S, 3S, and 4S), as well as the mean kinematic curves of the gait cycles recorded for each individual in each of the configurations tested (Additional file 1: Figure 10S).

#### SCI 1

Three gait configurations were recorded for this individual (Fig. 6): gait without stimulation (NS), gait with assistance in the standard mode in tibialis anterior, gastrocnemius and hamstrings muscles (NP TA GS H Standard) and gait with assistance in the standard mode in tibialis anterior and hamstrings muscles (NP TA H Standard), as shown in Fig. 6. The pulse trains used to assist were set up with up and down ramps to make the movement smoother (Table 1).

In Fig. 6, it can be seen that, for the second configuration, there was a significant decrease in knee flexion and dorsiflexion. In the experimentation itself, it was thought that the problem could come from assistance to gastrocnemius muscles, so such assistance was eliminated in the third configuration. This resulted in a decrease in plantarflexion at toe-off, but an increase in dorsiflexion at heel contact.

#### SCI 2

Three gait configurations were recorded for this user (Fig. 6): gait without stimulation (NS), gait with standard mode assistance in gastrocnemius muscles (NP GS Standard), and gait with standard mode assistance in both tibialis anterior and gastrocnemius muscles (NP TA GS Standard). Clinician team sought to improve dorsiflexion at mid-swing and heel contact, as well as plantarflexion at toe-off. They did not seek to improve knee flexion, so they did not assist in hamstrings muscles. The pulse trains used to assist were defined with up and down ramps for a smoother motion (Table 1).

In Fig. 6, it can be seen that, for the second configuration, there was a significant decrease in dorsiflexion at mid-swing and heel contact, as well as an increase in plantarflexion, on both sides (Additional file 1: Fig. 10S). The third configuration, resulted in some significant improvements in bilateral dorsiflexion in mid-swing and heel contact for both sides over the previous configuration, but only improved over the unassisted configuration on the right side. Plantarflexion also improved, especially on the left side.

#### Stroke 1

In this user’s test, four configurations were evaluated (Fig. 6): gait without stimulation (NS), gait with assistance in the cross mode for tibialis anterior, gastrocnemius and hamstrings muscles (NP TA GS H Cross), gait with assistance in the standard mode for tibialis anterior, gastrocnemius and hamstrings muscles (NP TA GS H Standard) and hybrid configuration with assistance in the standard mode (WH TA GS H Standard). The pulse trains used to assist were configured without up and down ramps for faster transitions (Table 1).

At the knee level, increased flexion was experienced in the swing, which was not significant between configurations. However, at the ankle level, increased dorsiflexion was experienced at mid-swing and ground contact for the configurations employing the NP independently, with no significant differences between configurations. Plantarflexion at toe-off decreased with respect to the unassisted configuration significantly for the stand-alone NP configurations without showing relevant differences between them. When comparing the hybrid configuration with respect to the unassisted configuration, no significant changes were evident.

#### Stroke 2

In the test, three configurations were recorded for this user (Fig. 6): gait without stimulation (NS), gait with WR assistance (WR) and gait with standard hybrid assistance (WH TA GS H Standard). The pulse trains used to assist were configured without up and down ramps to achieve faster transitions (Table 1).

The results show a significant increase in knee flexion in the swing due to WR performance. At the ankle joint level, an increase in plantarflexion was achieved for both assistance configurations, probably due to the walking posture with the WR. In addition, an increase in dorsiflexion was achieved, which reached a higher value for the hybrid condition, probably due to the assistance with the NP in the tibialis anterior.

## Discussion

With the increasing need for NPs adaptable to different contexts, applications and individual needs, as well as the ability to integrate with robotic devices to enhance rehabilitation, the main contribution of this study was the development of a novel modular NP system. The fundamental requirements common among systems reported in the literature were analyzed and essential requirements were established (Sect. 2.1). This served as the basis for the design and development of an NP system with modular architecture that has been characterized and detailed throughout this study. To validate it, it was adapted to a gait application in which it was combined with a knee-powered exoskeleton prototype. In addition, a technological personalization methodology based on clinical criteria was designed, allowing its successful use in individuals affected by SCI or stroke.

The architecture of this system is thoughtfully designed with modularity as its cornerstone. The primary objectives are flexibility, scalability, and personalization. Such type of modular system can be found in the literature [8, 15, 20–28]. Most of these systems offer the integration of some specific sensor types or limited third-party systems that provide some flexibility in upper and/or lower limb applications (Additional file 1: Table 1 S). However, we have successfully developed a system that maximizes flexibility by easily integrating a wide variety of sensors. It allows for the design of fully customized sensor networks tailored to specific conditions. This has been achieved through the integration of Biometrics Ltd.‘s third party system offering wired or wireless sensors, which also translates into great portability and ease of adaptation to the needs of any context, application and individual. However, it is not limited to this system alone, as the features offered by the CU allow for easy integration of stand-alone sensors or other systems (e.g., knee-powered exoskeleton prototype developed by ABLE Human Motion). Furthermore, this flexibility allows the system to adapt to unforeseen events, such as the failure of a sensor (e.g., Additional file 1: Stroke 2 case Fig. 11S), and the sensor network can be reorganized easily and quickly as required.

As for the stimulation nodes, the number of channels of the stimulation nodes of the modular systems in the literature varies between 1 and 4, which is in accordance with our development and is sufficient for many applications. However, thanks to the scalability of the system, it is possible to increase the number of channels as needed for different case scenarios. These nodes use transcutaneous electrodes, like most of the current systems (Additional file 1: Table 1S). One of the main advantages of systems employing transcutaneous electrodes over those employing implanted electrodes is that these can be more easily readapted to the changing needs of the user during rehabilitation. Moreover, implanted electrode systems usually require a surgery with additional risks for the subject. Compared to systems reported in the literature, the ES nodes presented in this work allow for versatile communication modes that can be used depending on the application and context. The battery autonomy of the developed ES node far exceeds the battery autonomy reported in similar systems reported in the literature (Additional file 1: Table 1S). Moreover, it is the only modular NP system designed with an external battery that can be replaced, allowing continued use of the device. In this regard, reducing the energy storage capacity of the battery could be analyzed. This would help to reduce the dimensions and weight of each node, the latter being comparable to the stimulator of similar characteristics Compex Motion [8]. The dimensions are the feature that needs major improvement because it is a prototype in which the spatial organization was not sought to be optimized (Additional file 1: Table 1S). This 3D encapsulation allowed simple assembly in different contexts, but could be optimized to reduce the actual dimensions.

The ES parameters that allow varying ES nodes compared to the other systems reported in the literature are similar in amplitude, although there is more variability in pulse width and frequency (Additional file 1: Table 1S). However, our system offers greater flexibility in terms of pulse phase (Additional file 1: Table 1S). Regarding the pulse train, all systems present very similar characteristics, with the trapezoidal pulse train being the most widespread (Additional file 1: Table 1S). In our case, the system offers some personalization of the trapezoidal pulse trains, as do Popovic et al. [8] and Cerone et al. [15, 27].

Regarding the CU, this system presents a similar structure to most of the systems found in the literature, where it acts as a coordinator and communicates with a personal computer for GUI deployment. Other systems have opted for the use of a stimulator as a coordinator of the stimulation [8] or for a decentralized architecture as is the case of Andreu et al. [21] and Cerone et al. [15, 27]. No advantages of one type of architecture over the rest have been found and all meet the established requirements of flexibility, scalability and personalization.

The CU used in this system offers a higher connection and communication capacity, which allows the combination with other devices in a simple way, as is the case of robotic devices, and to apply different assistance strategies (sequential, synchronous or asynchronous assistance, or interference strategies) [10, 36, 37]. The only system mentioned that was also designed for this purpose was the one proposed by Qu et al. [22, 26]. However, they did not present any integration with this type of technology or validation in this regard. On the contrary, our system has proven to be able to be combined with this type of technology in a satisfactory way. This has been demonstrated at the physical level, where 3D printing has played a fundamental role in achieving a simple, fast, safe and functional integration. It has also been demonstrated at the software level, where the NP system has integrated the WR actuation mode to create a simple open-loop hybrid control achieving coordinated joint assistance.

In terms of the way of interacting with the system, an intuitive GUI has been developed in Python, a language widely used in research and the health field, with the aim of being able to extend the functionalities offered in a simple and fast way according to the needs. This interface allows the configuration of the system and all its features to adapt it to each context, application and individual’s needs. In addition, it adds calibration functionalities that may be of interest in clinical practice to achieve a more objective and precise adjustment, which have not been observed in the other systems reviewed. This GUI also facilitates the creation of stimulation protocols and preset configurations that can be loaded quickly reducing setup times, just like the system proposed by Popovic et al. [8]. In addition, it allows data capture, logging and visualization to improve system performance and evaluate the assisted training performed. These functionalities not only gather most of the functionalities offered by the systems reported in the literature, but extend them to achieve a more precise and objective personalization (automatic calibration) or a more agile configuration that does not limit the effective time of the therapy session (loading of preset configurations with respect to the last session).

Regarding safety, our system, like systems reported in the literature, integrates stimulation nodes that operate within safe limits and have galvanically isolated ES channels. Moreover, batteries are used to power them, which reduces the risk of possible leakage currents affecting the user, as in the case of grid-connected systems [15]. It should be noted that immediate ES shutdown mechanisms are proposed in the literature through individual switches per stimulator [8, 15] or fault detection systems that cause the shutdown of the ES [8, 15, 21, 22, 26]. However, it is considered crucial to be able to stop stimulation jointly in a scalable system. Despite this, our system is the only one that implements a physical joint stop, in addition, to the corresponding node-specific ON/OFF switch. At the software level, the GUI provides guidance information for the use of the system, and also integrates fail-safety and fault logging for continuous optimization of the modular NP system.

The system has been validated in a gait application for which a gait event detection algorithm and four open-loop control modes based on a state machine have been implemented. This allowed to assist in the preset gait phases in an appropriate way, as can be seen in Fig. 10S S of additional file 1. Closed-loop control strategies can also be implemented that are executed both in the CU and in an external system connected and communicated through the implemented resources. The pilot validation study performed demonstrated the NP can be adapted in real-time to the functional requirements of patients and clinical decisions of the therapists.

The literature offers a variety of FES application strategies, resulting in diverse outcomes. This variability stems from applying FES to different target populations, combinations of muscle groups, and timing variations during gait, leading to a broad spectrum of assistance approaches [61, 78–83]. This diversity of approaches highlights the need for further research analyzing the immediate impact of FES assistance. Additionally, there is a need for studies that aid in identifying target muscle groups and phases of assistance. Despite the complexity of comparing with other findings in the literature, Kesar et al. noted that dorsiflexion is not as pronounced when ankle plantarflexors are assisted along with gastrocnemius stimulation compared to when only ankle dorsiflexors are assisted [61]. A similar trend was observed in SCI 1 when comparing configurations 2 and 3, where an increase in dorsiflexion during mid-swing and contact was evident upon removal of gastrocnemius stimulation (Fig. 6). Additionally, Chen et al. demonstrated that inadequate takeoff during the push-off phase is linked to reduced ankle and knee dorsiflexion in swing due to a decrease in forward propulsive force [84]. We believe that this observation may align with the case observed in SCI 1 (Fig. 6), where inadequate plantarflexion during push-off led to difficulties in executing the step effectively. Consequently, knee flexion during mid-swing and dorsiflexion during swing decreased for the second configuration (Fig. 6). As a result, the clinical team opted to introduce assistance in the tibialis anterior, resulting in improved dorsiflexion and enhanced patient comfort. In SCI 1, SCI 2 and Stroke 2, the last configuration employed achieved a more beneficial impact than the previous configuration, highlighting the importance of performing iterative adjustment and the importance of clinical judgment.

At the knee level, no differences were observed between the stand-alone or hybrid WR mode (Stroke 1 and Stroke 2 cases Fig. 6). This is because an open-loop control system has its limitations, and in hybrid systems, it is necessary to address the challenges introduced by hybrid actuation control, such as actuation redundancy [35, 85–88]. The implemented open-loop strategies represent a simpler approach that does not account for the mentioned redundancy occurring only at the knee level. Therefore, in this joint the flexion angle is mainly governed by the mode of operation of the WR which follows a preset trajectory. However, in the Stroke 2 case, where a WR configuration versus a hybrid configuration can be compared, an increase of swing dorsiflexion on the ankle unique to the modular NP is observed due to the lack of redundancy. Finally, it should be noted that an overall increase in hip flexion in Stroke 1 and Stroke 2 individuals was evident in the kinematics (Additional file 1: Fig. 11S). This may be due to the individual’s own posture when walking with the WR, which was characterized by a forward lean. In fact, Lewis et al. observed that a forward leaning position during walking leads to a more flexed hip throughout the gait cycle [89]. This could also have been the reason why in the hybrid configurations a dorsiflexion is not observed as pronounced as in the configuration with the modular NP (Stroke 1).

In this study, technical and experimental limitations have been identified that should be considered for future developments and work. In the context of FES, this study employs an open-loop control system based on a state machine, where the assistance is pre-set, allowing only minor temporal variations in gait pre-swing. Different temporal stimulation protocols have been tested in the literature and even clinical stimulation targets have been established for different muscle groups, but it is still not clear which is the optimal way to apply FES within the gait cycle [83, 90, 91]. In this regard, future developments need to integrate the ability to personalize stimulation at the temporal level within the gait cycle. In this way, it will be possible to adapt the stimulation pattern to achieve greater personalization. In addition, a limitation of this study is that we did not get to test the closed loop due to time constraints for implementation. Therefore, future studies need to integrate closed-loop control algorithms that allow real-time adjustment of assistance based on the needs of the individual and that consider changes in muscle response and fatigue. In this way, it will be possible to achieve a greater capacity for personalization adapted to the context and the individual, simplifying the clinical work of configuration and streamlining the process of technological personalization.

Trapezoidal pulse trains have not been shown to be optimal either, although they are the most widespread and widely used. Therefore, it would be interesting to provide greater flexibility in the formation of tailored pulse trains [9, 92]. In this sense, the implementation of assisting modes of operation based on volitional capabilities of the individual should also be considered. Another drawback to be considered in the use of superficial electrodes is the difficulty in stimulating the deep musculature. This limitation made it difficult to assist at hip level, so that assistance was not used in that joint with the tensor fasciae latae muscle group was not used. In this regard, some studies have proposed the use of interference strategies for stimulation of the deep musculature [36, 37], so it would be interesting to consider increasing the stimulation frequency offered by each stimulation node in order to apply this type of strategy. An alternative strategy to improve hip flexion could be to take advantage of the withdrawal reflex. Del-Ama et al. investigated this reflex’s potential in combination with the Lokomat in a pilot study involving two SCI patients [93], although further work is needed. However, using this strategy carries a drawback: the uncontrolled nature of the movement, which may pose risks of instability. Hence, we do not yet consider it a safe option for use on the floor with a WR. Nonetheless, for potential future investigations, we propose integrating our modular NP system with static robotic devices like the Lokomat, which provide patient stability. Alternatively, it could be combined with robotic ambulation devices similar to those used in our study, albeit supplemented with a robust safety system such as a mobile crane.

Moreover, some studies have shown that co-modulation of two or more ES parameters can be more beneficial in therapy than modulation of a single parameter [19], so the functionality of the system should be increased in this sense to favor research with this type of techniques. The use of different pulse waveforms has also been discussed in the literature, so it would be positive to introduce more flexibility by adding other waveforms of interest [94].

Regarding experimentation and gait adaptation, the gait adapted NP has employed a gait event detection algorithm based on kinematic information. Due to the heterogeneity of joint motion that can be found among different individuals, other gait segmentation methods need to be explored in the future, in order to be able to select the most suitable one according to the selected sensors and the capabilities of the particular individual. Another limitation that has been detected was the need to use 2 GUIs for the control of the devices that were combined (Fig. 4). In the future, it would be interesting if both systems could be combined under the same GUI to facilitate clinical practice. Furthermore, in relation to the combination with robotic devices, it is necessary to develop modular robotic devices with similar characteristics to those implemented in this modular NP system. This will favor the development of even more personalized strategies to enhance assistance with the selection of the optimal technological configuration. In future work, the implementation of hybrid strategies will require the development of control algorithms addressing the challenges of hybrid control to optimize the assistance provided by each system.

Finally, this study has allowed us to perform a pilot evaluation of the system with four users. In the future it is necessary to conduct more studies that include individuals with other assistance needs to continue advancing in the identification of personalization needs to be implemented in this type of systems. It is essential to integrate the requirements and technical advancements of these assistive systems into a personalization protocol, enabling the clinical team to achieve accurate and tailored configurations based on individual needs. Procedurally, calibration remains a complex process with a subjective component. The usefulness of objective calibration methods such as the one implemented in this system needs to be investigated in order to integrate it satisfactorily within the personalization procedure.

## Conclusion

This study has analyzed the need for the development of NPs that provide greater personalization capabilities and that can be combined with robotic assistive devices. Other systems reported in the literature have been analyzed and fundamental requirements for consideration were identified.

The new modular NP suitable for hybrid FES-robot applications integrates a number of features that make it a convenient device to further advance the quest to maximize the benefits of NP and WR technologies in clinical rehabilitation. Moreover, an application-oriented adaptation has been implemented during gait activity and a configuration protocol has been developed for technological personalization based on the needs of the individual. As a result, it has been possible to validate it in an experimental test with four users, which has yielded kinematic results comparable to those reported in the literature. This experimental test not only demonstrated the versatility of the new system in creating hybrid solutions but also showcased its ability to adapt to the clinical requirements of individuals with different neurological conditions, specifically spinal cord injury (SCI) and stroke.

## Electronic supplementary material

Below is the link to the electronic supplementary material.

Supplementary Material 1

## Data Availability

No datasets were generated or analysed during the current study.

## Abbreviations

Ch: Channel
CT: Comfort threshold
CU: Control unit
drd: Down ramp duration
ES: Electrical stimulation
EMG: Electromyographic or Electromyography
F: Female
FAC: Functional Ambulation Categories
FES: Functional electrical stimulation
GS: Gastrocnemius
G: Gender
GM: Gluteus maximus
GUI: Graphical user interface
H: Hamstrings
HC: Heel contact
HO: Heel off
He: Height
I: Individual
A_L_: Left ankle
H_L_: Left hip
K_L_: Left knee
αK_L_: Left knee angle
M: Male
K1: Maximum knee flexion during swing
MG: Muscular group
NP: Neuroprosthesis
Q: Quadriceps
ROM: Range of motion
A_R_: Right ankle
H_R_: Right hip
K_R_: Right knee
αK_R_: Right knee angle
SCI: Spinal cord injury
TFL: Tensor fasciae latae
TA: Tibialis anterior
T: Time
TO: Toe off
urd: Up ramp duration
WISCII: Walking Index for Spinal Cord Injury
WH: Wearable hybrid
WR: Wearable robot
W: Weight
