# Supplementary material for: A new modular neuroprosthesis suitable for hybrid FES-robot applications and tailored assistance
Source: J Neuroeng Rehabil. 2024 Sep 4;21:153. doi: 10.1186/s12984-024-01450-6 (PMC11373245; doi:10.1186/s12984-024-01450-6)
Supplement: Supplementary file 1 — Supplementary Material 1 [file 12984_2024_1450_MOESM1_ESM.docx]

# **Supplementary Material**

# **1. Technical specifications of our modular NP system**

## 1.1 Sensors

An external third-party system from the company Biometrics Ltd (Newport, UK) has been integrated and is deployed on a 64-bit Windows operating system. This system allows the use of a wide variety of wired or wireless sensors with integrated batteries, such as contact switches, force sensors, pressure sensors, accelerometers, electrogoniometers, EMG sensors, and torque meters, among others (Fig. 1 A) (1). These sensors have small dimensions, weigh between 5 and 30 g, and the wireless versions have an autonomy of up to 12 h and low battery warning indicators. The system also offers an 80 g trigger that can be used as a manual trigger or to synchronize systems. The latter is very useful when external recording systems are used in the realization of surveys, such as a photogrammetry system (VICON, BTS, CODA...). In addition, from a dedicated GUI, the sensor network can be flexibly configured and data from up to 24 channels can be logged. The data provided by the sensor network can be used indirectly without the need for the software provided by Biometrics Ltd, as the company itself provides dynamic link libraries (DLLs) with functions that facilitate this. In this way, data related to measurements, sensor configuration and errors that may occur during sensor use can be accessed. Sensor integration is not limited to this third-party system, but other sensors can be easily integrated via wired or wireless connection thanks to the features of the selected control unit detailed later in this section (Fig. 1 A).

## 1.2 Nodes of ES

The ES units or stimulation nodes consist of an external battery, a power stage, a control stage, 4 galvanically isolated electrical stimulation channels and a communication module (Fig. 1 B). The node has a USB A connection for the connection of an interchangeable external battery (Fig. 1 B). For this prototype, a 10000 mAh battery (Power Bank DCU 10000 mAh Dual output (2)) with remaining charge LED indicators and intelligent overcharge, short-circuit and overheat protection has been selected. In addition, being interchangeable, continuous use is favored, so spare batteries were available. The power stage receives power from the external battery and is responsible for generating and delivering the necessary current to the control stage (Fig. 1 B). The control stage integrates an ATmega128L microcontroller and is where the firmware has been implemented to adjust and manage the different parameters of the ES delivered by each of the stimulation channels (Fig. 1 B). Stimulation with a controlled, stable and precise current is achieved by a Howland circuit (3). The communication module is responsible for the bidirectional communication between the peripheral nodes and the CU (Fig. 1 B). The connection can be wired or wireless (Wireless Personal Area Network based on the IEEE 802.15.1 standard or Bluetooth) and allows transmitting the system status and receiving adjustment and configuration commands. In addition, each stimulation node has four stimulation channels and an on/off rocker switch (Fig. 1 B). They also have several LEDs that show the status of the device (on/off, stimulating/not stimulating) (Fig. 1 B and Fig. 1S A of the supplementary material). As for their performance characteristics, they meet the performance requirements found in the literature (4–6) and the main characteristics can be found in Table 1S of the supplementary material. The node was encapsulated by 3D printing so that the battery could be easily replaced and even the electronics could be accessed (Fig. 1S A of the supplementary material). In addition, a female-type quick-release buckle fastening structure was designed with the aim of being able to quickly create 3D fastening parts with male-type quick-release buckle structure that would allow the NP to be assembled on any surface (Fig. 1S B of the supplementary material).

## 1.3 Control Unit

For the CU, a LattePanda Alpha 864 was selected, which is a lightweight (120 g) development board often used in prototyping and embedded system development projects (Fig. 1 A). This versatile development board can be adapted to different applications and has an Intel Core m3 processor that provides sufficient processing power for performing complex calculations and executing control algorithms. It is compatible with operating systems such as Windows 10 and Linux, which facilitates the development and execution of control software, as well as the development of a GUI that simplifies user interaction and configuration. It offers various connectivity options (USB ports, USB C, HDMI, Bluetooth, Wi-Fi, Ethernet...) and communication interfaces (GPIO, UART, I2C...) that facilitate connection to other system components and even third-party systems such as robotic devices (Fig. 1 A). In addition, it has a compact form factor and, although it can be powered by a mains connection, it is possible to power it using an external battery, which facilitates portability and integration into wearable systems. It also has an active community and technical support that can promote development and maintenance. The LattePanda Alpha 864 was encapsulated using 3D printing so that the battery could be easily replaced and even the electronics could be accessed (Fig. 2S of the supplementary material). This encapsulation was provided with a structure that would later allow it to be assembled to a robotic device secured with a "T" piece (Fig. 2S of the supplementary material). The external battery chosen was the "Coolbox P/N: COO-PB20K-PD45 Type-C power bank".

## 1.4 GUI

At the software level, the system is associated with an intuitive GUI developed in Python because it is an interpreted language that facilitates the rapid development of prototypes compatible with various platforms (Windows, Linux, macOS). In addition, it is a language widely used in scientific research and medical applications that has a large community, resources and a wide variety of graphic libraries that speed up the development of attractive and functional visual interfaces. In addition, it is easy to implement new functionalities in the software, if deemed necessary.

## 1.5 Modular NP applied to gait

For the described context, a sensor network consisting of a set of up to 6 dual-axis wireless electrogoniometers (Biometrics Ltd, Newport, UK) was configured to measure the angular motion of each of the lower limb joints during gait. Type W110 electrogoniometers were selected for ankles and type W150 for hips and knees (Fig. 3 A). These sensors are capable of measuring angles up to 180°, with a resolution of 0.1°, in two orthogonal axes, send the information in real time at 100Hz and work at a distance of up to 30m to the USB receiver (1). In addition, angular information is a type of data that is widely used in the development of closed-loop control algorithms, so it was determined that it could be of great use in this context.

A certified emergency button with a rotational release system (XW1E-BV404M-R (7) from IDEC) capable of controlling up to 4 different lines was chosen (Fig. 3 A). This emergency button was encapsulated by 3D printing and provided with a female-type quick-release buckle structure like that of the stimulation nodes in order to be able to use the same male-type attachment parts and to facilitate the development of new 3D attachment parts if necessary (Fig. 1S B and Fig. 1S C of the supplementary material).

# **2. Comparison of technical specifications**

|  | **Loeb et al. & Weber et al.** (8,9) | **Popovich et al.** (10) | **Qu et al.** (11,12) | **Jovitic et al.** (13) | **Andreu et al.** (14) | **Cerone et al.** (15) | **Kilgore et al.** (16)**, Makowski et al.** (17) | **Our system** |
| --- | --- | --- | --- | --- | --- | --- | --- | --- |
| **Sensors** | EMG, inertial | EMG, EMG system and a gait event detection system (gyroscope and 3 pressure sensors) | EMG | Inertial and pressure sensors | EMG, inertial, pressure sensors, goniometers | EMG, goniometers | EMG, inertials, temperature | Contact switches, force sensors, accelerometers, electrogoniometers, EMG, torsiometers, trigger and others |
| **Channels per stimulator** | 1 | 4 | 2 | 2 | 2 | 1 | 4 | 4 |
| **Pulse phase** | Monophasic | Monophasic or biphasic symmetric | Biphasic symmetric | - | Biphasic | Monophasic or biphasic | Biphasic symmetric | Monophasic or biphasic symmetric or biphasic asymmetric |
| **Pulse amplitude (resolution) [mA]** | 0 to 3 (0.19)  3 to 30 (1.69) | 0 to 125 (1) | 0 to 50 (0.10) | 0 to 70 (0.07) | 0 to 100 (0.10) | 0.1 to 100 (0.10) | 0-20 (0.10) | 0 to 90 (0.35) |
| **Pulse width (resolution) [us]** | 4 to 512 (1.98) | 0 to 16000 (500) | 0 to 500 (1) | 10 to 1000 (3.75) | 50 to unknown (1) | 0.01 to 100 (0.01) | 1 to 255 (1) | 250 to 500 (2.4) |
| **Frequency (resolution) [Hz]** | 16 to 50 (-) | 1 to 100 (1) | 8 to 1000 (1) | - | 1 to 1000 (-) | 0.1 to 200 (0.1) | 1 to 50 (<3Hz) | 20 to 100 (1) |
| **Pulse train** | Trapezoidal | Tailored manually or with an input signal | Tailored manually | Trapezoidal | Trapezoidal | Tailored manually or with an input signal | - | Trapezoidal and manually tailored |
| **Battery autonomy [h]** | - | 8 | - | - | 4 a 11 | 13 | - | 33 |
| **Stimulator weight with battery [g]** | < 1 | 420 | - | 45 | 98 | 350 | - | 480 |
| **Stimulator dimensions [cm^3^]** | 0,05 | 356 | - | 53 | 132 | 352 | ≈ 4 | 1127 |
| **Electrodes** | Implanted | Transcutaneous | Implanted and transcutaneous | Transcutaneous | Transcutaneous | Transcutaneous | Implanted | Transcutaneous |
| **Component connection** | Wireless | Wired | Wired | Wired and wireless | Wireless | Wireless | Wired | Wired / Wired and wireless / wireless |

Table 1S. Technical specifications of modular NP systems. This table shows the technical specifications of our system and the systems considered in the literature.

**3. 3D parts and enclosures**

This section shows all the figures related to the 3D encapsulation of the control unit and electrostimulation nodes that form the modular NP system. Also shown are the parts for donning/doffing the modular NP system and the hybrid assembly in combination with the WR (knee-powered exoskeleton prototype developed by ABLE Human Motion).


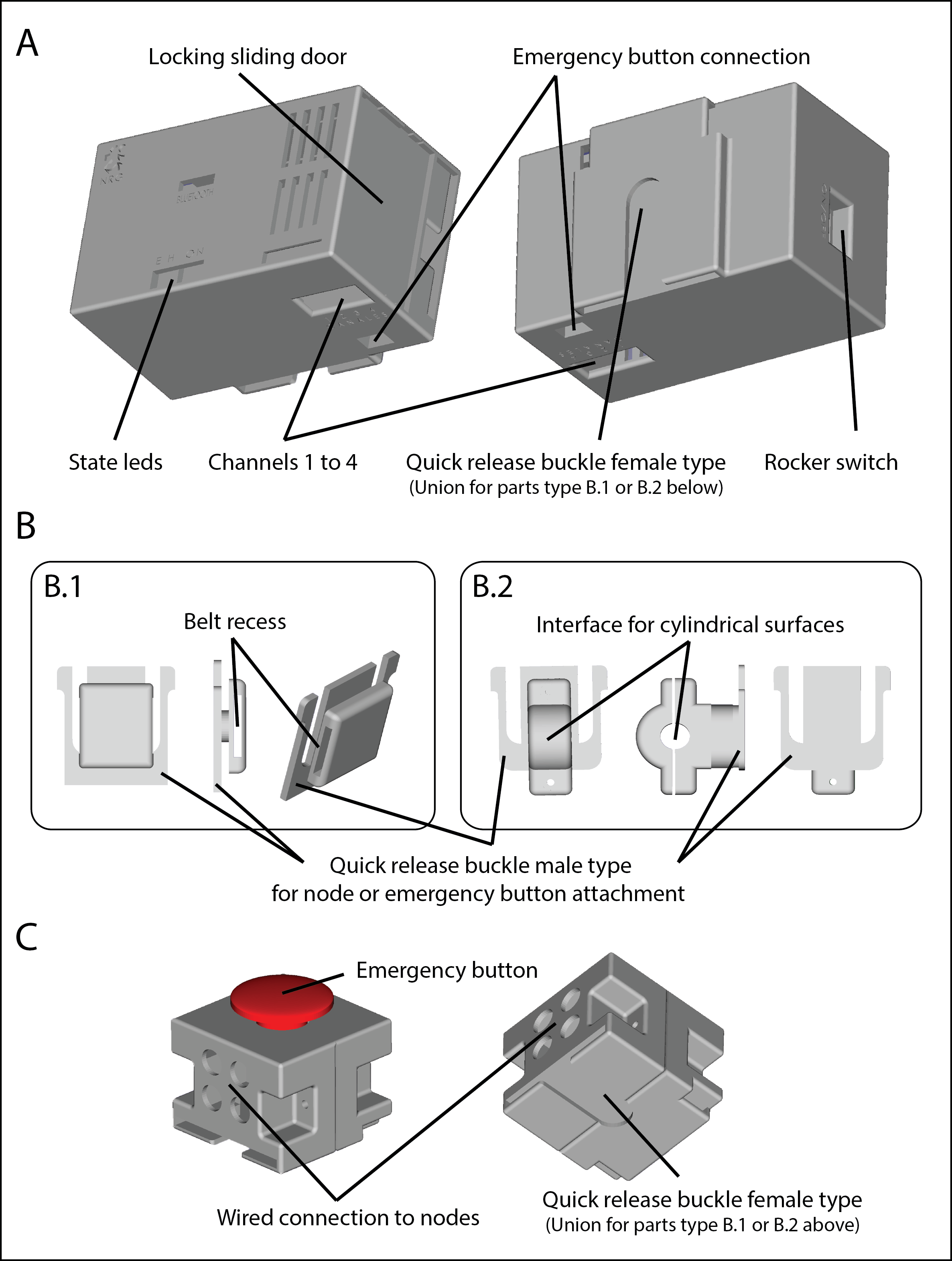


Fig. 1S. (A) Stimulation node, (B) restraints and (C) emergency button. Section B shows the restraints used to attach the stimulation node or emergency button to a belt (B.1) or to a cylindrical surface (B.2).


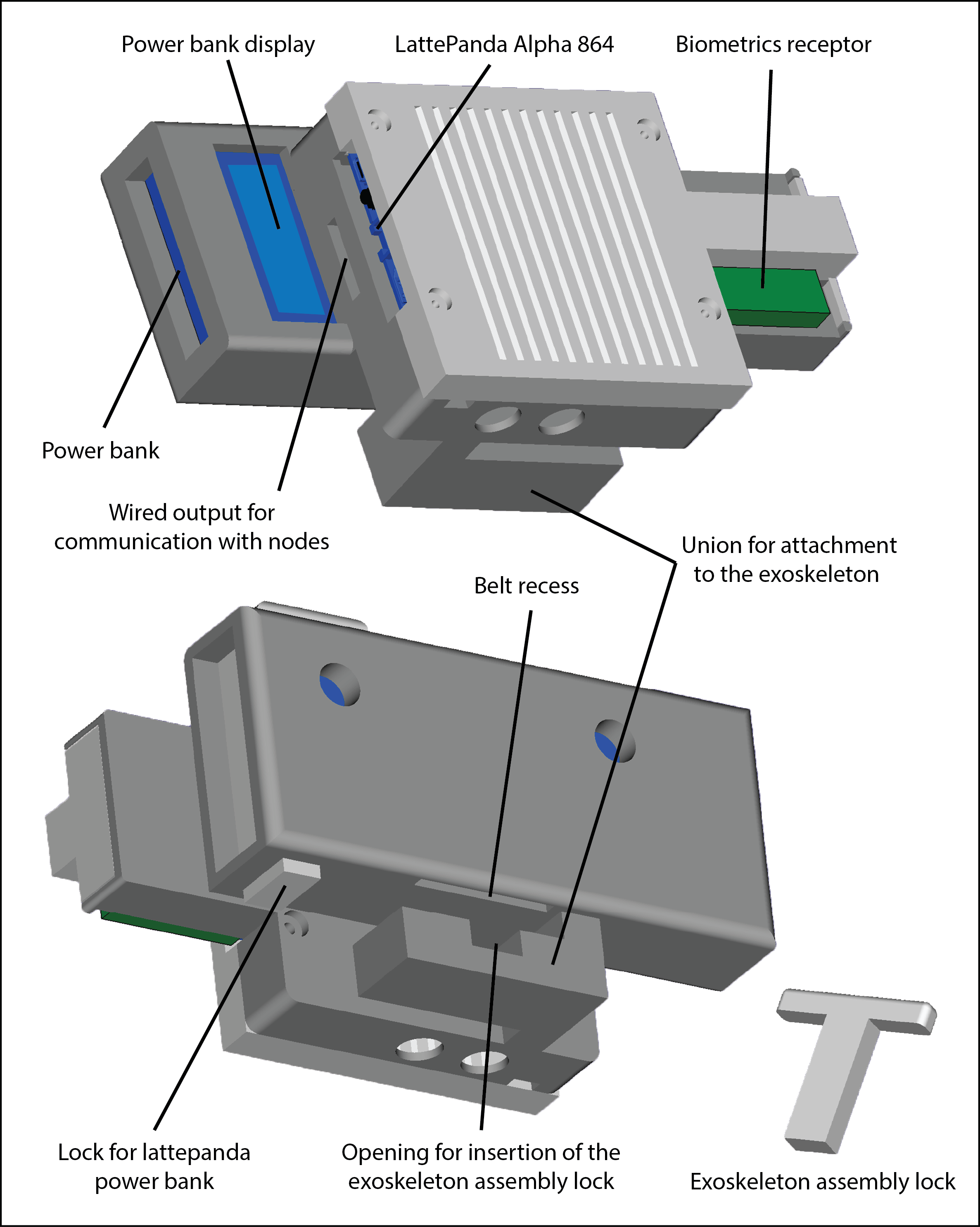


Fig. 2S. Control Unit: details of the 3D enclosure and the different elements of the control unit are shown, as well as the part used to secure it to the central rear part of the WR (Fig. 8S).


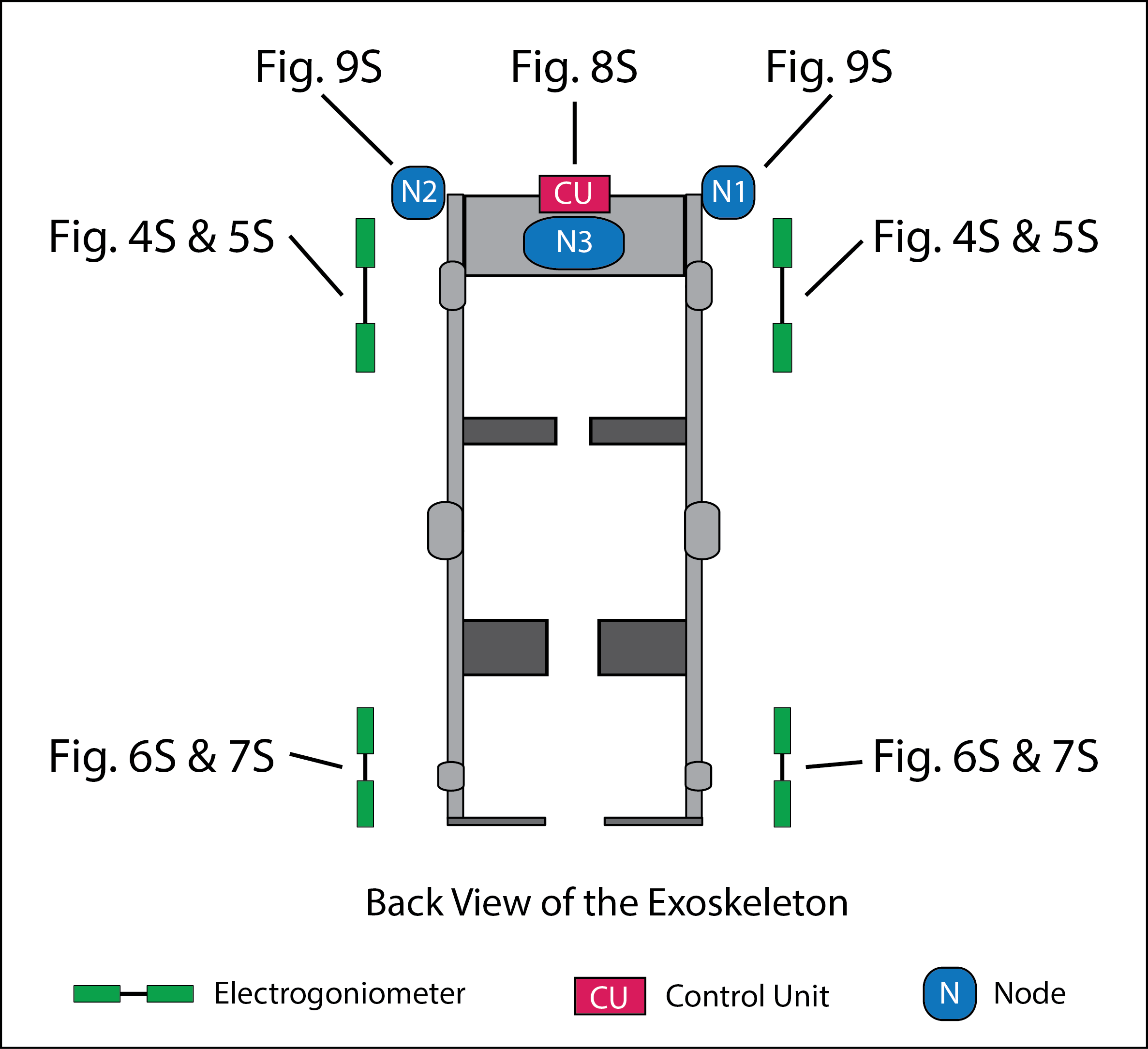


Fig. 3S Hybrid donning/doffing. This configuration only requires electrogoniometers located in the hip and ankle because in the knee the WR itself provides us with the joint angle in real time. Special 3D parts adjusted to the WR are used to place the electrogoniometers in a precise and standardized way, without hindering the donning of the WR or impairing its performance. The nodes and control unit are located where indicated in the figure. Key points of interest and associated figures have been highlighted, where additional details on the donning process can be found. The placement of the emergency button is essential that it be accessible to facilitate quick access to it if needed. It was determined that it should be placed on the belt as in the stand-alone modular NP system at the front, so it does not appear in the diagram.


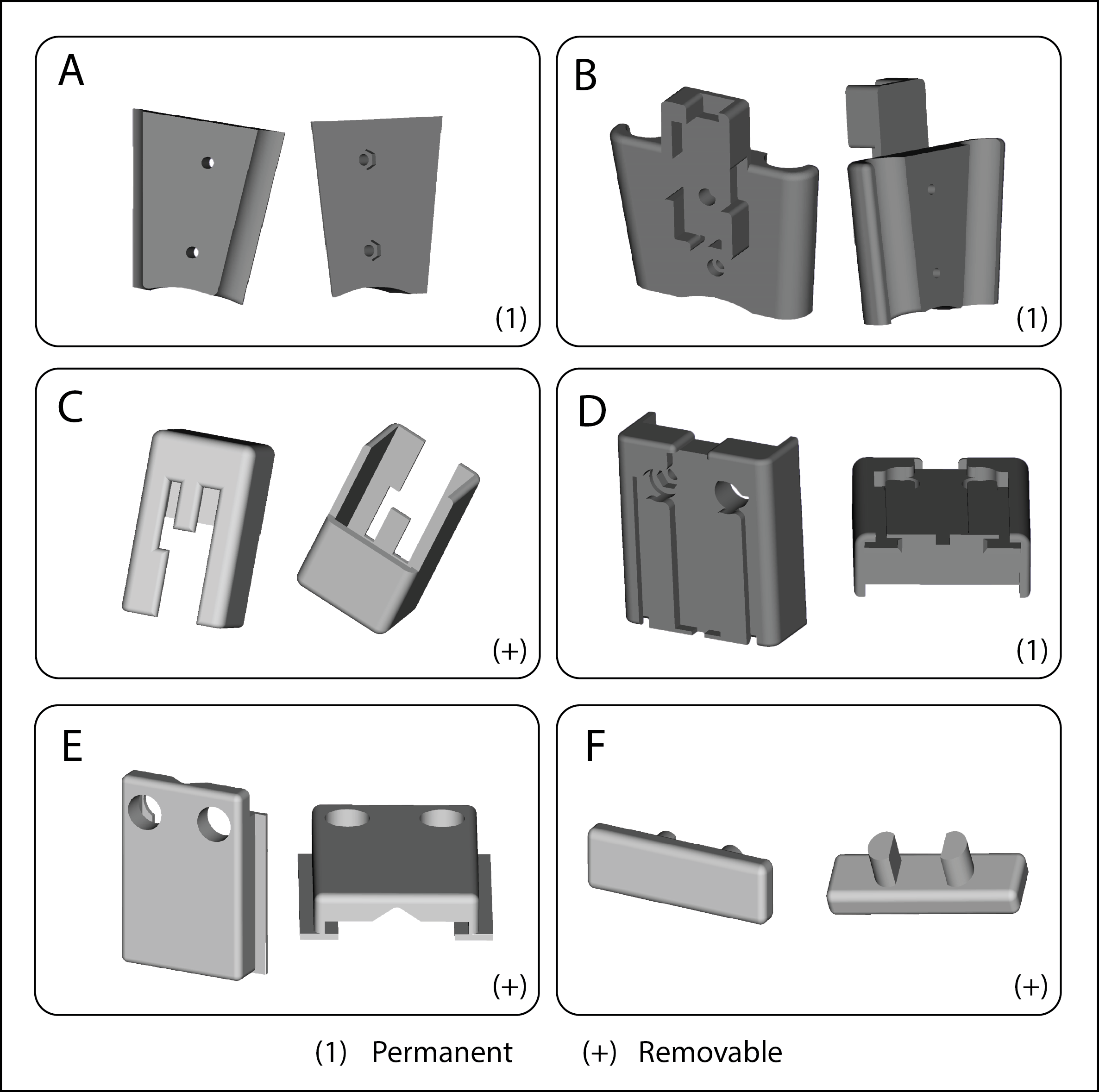


Fig. 4S Electrogoniometer hip assembly pieces. These parts are used to assemble the hip electrogoniometer to the WR, a process detailed in Fig. 5S. It details the parts that only need to be assembled once and those that should be removed during the donning/doffing process. Pieces A and B are used as clamps that are attached above the center of rotation of the hip’s WR and allow the fixation of the upper part of the electrogoniometer, which is secured with piece C. Part D is positioned below the center of rotation of the hip’s WR and allows the lower part of the goniometer to be fixed. Part E slides along the rails of part D to secure the electrogoniometer and piece F is used to lock the structure formed by parts E and D.


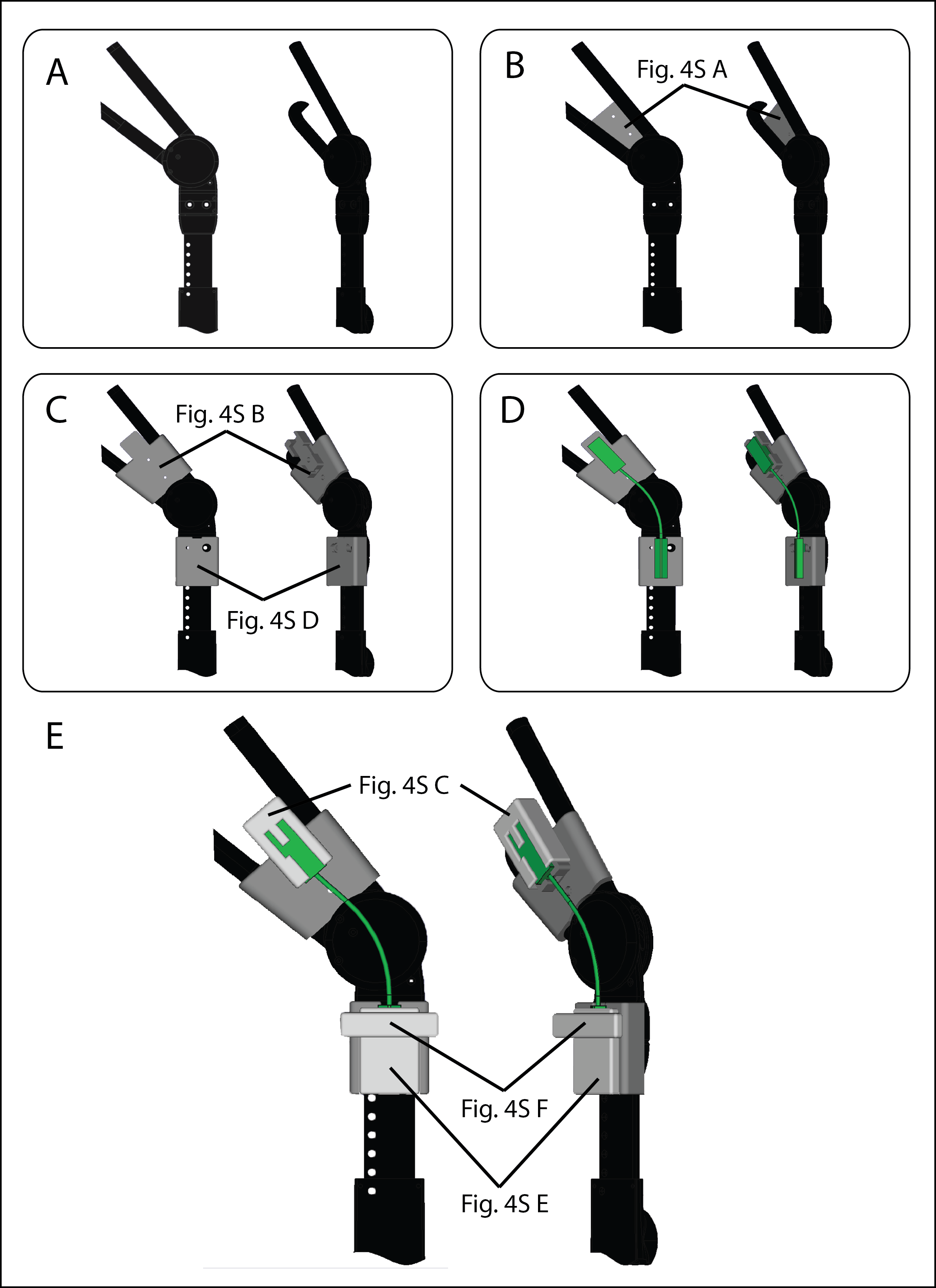


Fig. 5S. Electrogoniometer hip assembly. The assembly process is detailed step by step, being the first step A and the last step E. The parts used have been previously detailed in Fig. 4S. Section A shows only the hip’s WR. Section B adds part Fig. 4S A. Section C adds parts Fig. 4S B and Fig. 4S D. The above steps only need to be performed the first time the hybrid is assembled. The rest of the steps will need to be repeated during the donning/doffing process. Section D adds the electrogoniometer (in green) embedded in parts Fig. 4S B and Fig. 4S D. Section E shows the electrogoniometer secured with parts Fig. 4S C, Fig. 4S E, and Fig. 4S F.


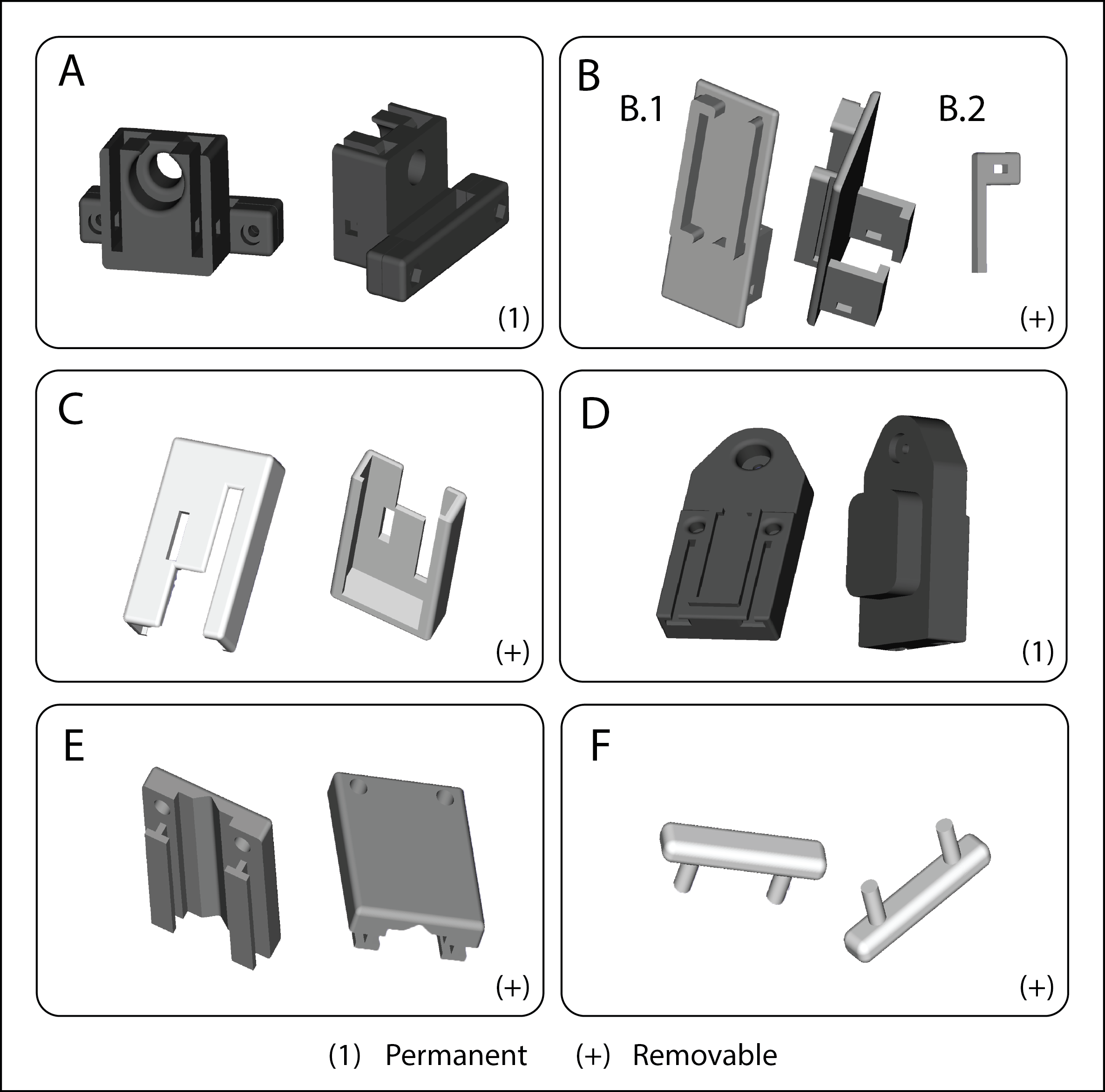


Figure 6S. Electrogoniometer ankle assembly pieces. These parts are used to assemble the ankle electrogoniometer to the WR, a process detailed in Fig. 7S. It details the parts that only need to be assembled once and those that should be removed during the donning/doffing process. Part A is used as clamps that are attached above the center of rotation of the ankle. Part B.1 slides along the rails of part A and B.2 is used to lock it. On B.1 the upper part of the electrogoniometer is fixed, which is secured with part C. Figure D is placed below the center of rotation of the ankle and allows the lower part of the electro direction finder to be fixed. Part E slides along the rails of part D to secure the electrogoniometer and part F is used to lock the structure formed by parts E and D.


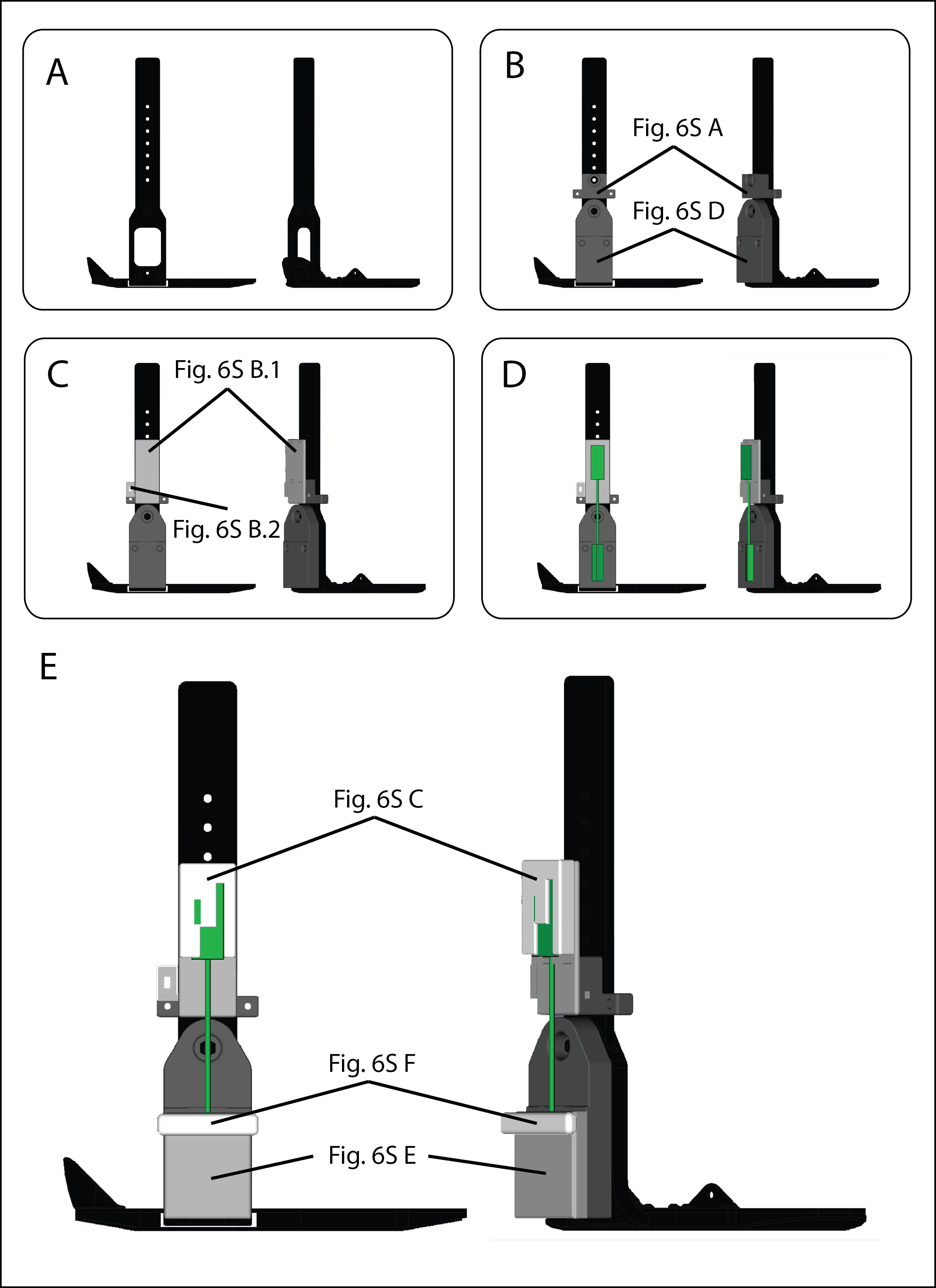


Figure 7S. Electrogoniometer ankle assembly. The assembly process is detailed step by step, with the first step being A and the last step being E. The parts used have been previously detailed in Fig. 6S. Section A shows only the ankle of the WR. Section B adds parts Fig. 6S A and Fig. 6S D. The above steps only need to be performed the first time the hybrid is assembled. The rest of the steps will need to be repeated during the donning/doffing process. Section C adds parts Fig. 6S B1 and Fig. 6S B2. Section D adds the electrogoniometer (in green) fitted to parts Fig. 6S B.1 and Fig. 6S D. Section E shows the electrogoniometer secured with parts Fig. 6S C, Fig. 6S E and Fig. 6S F.


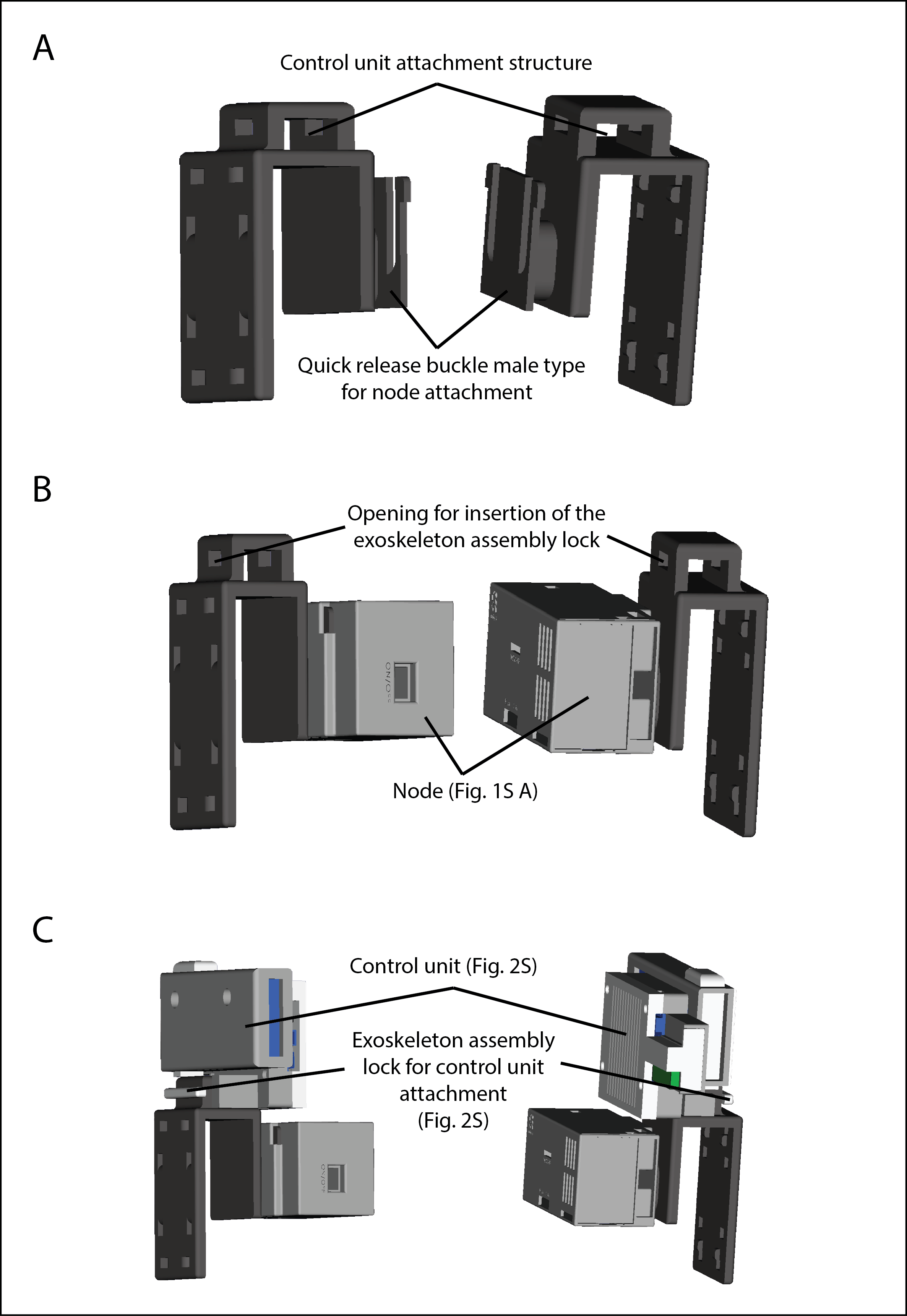


Fig. 8S. Central attachment. This figure shows the central attachment piece that can remain in place in the WR once installed. In addition, the figure shows the placement of a stimulation node (Fig. 1S A) through a male type attachment similar to Fig. 1S B and the control unit (Fig. 2S). The latter is secured with the exoskeleton assembly lock (Fig. 2S).


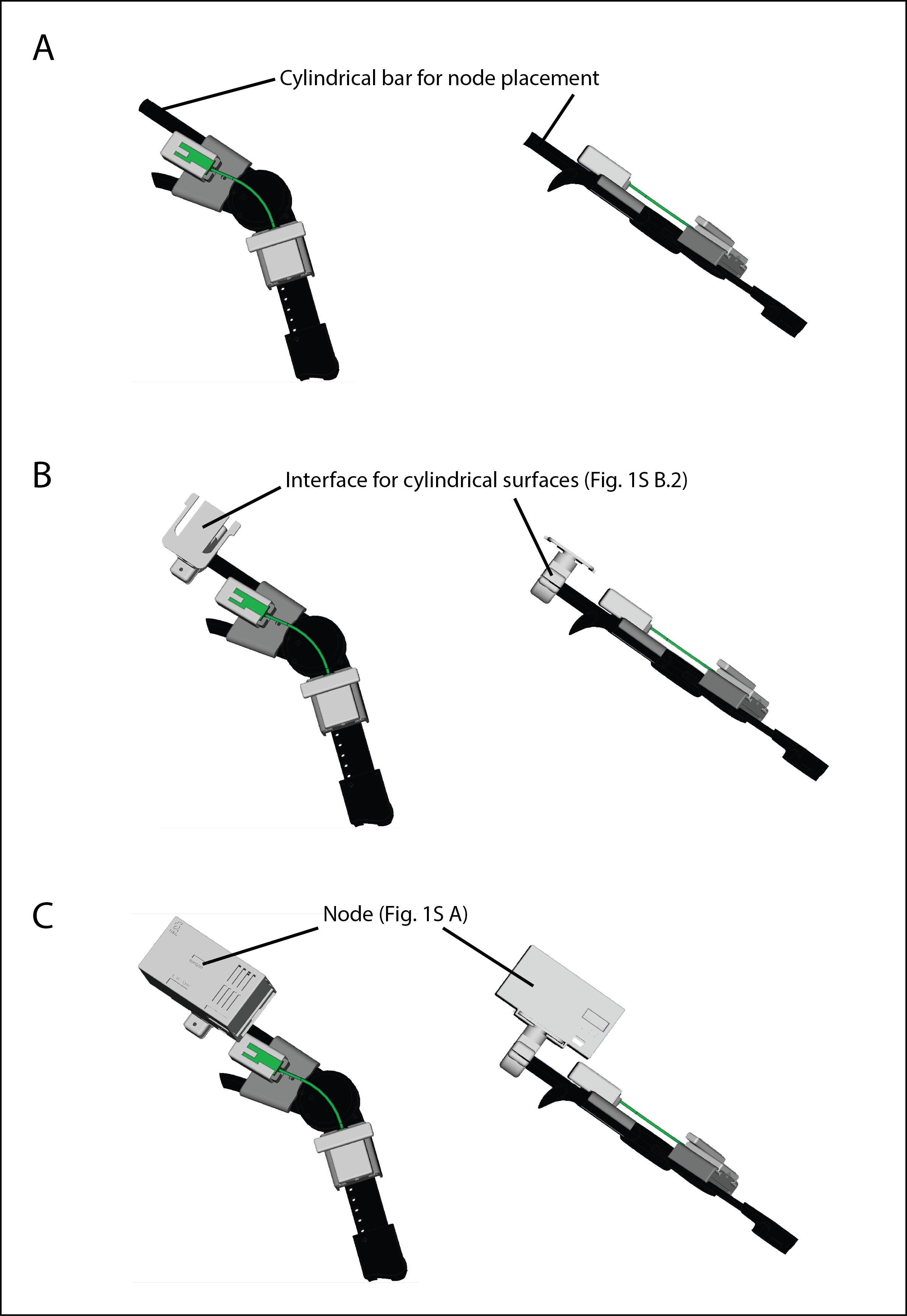


Fig. 9S. Lateral attachment. This figure shows the attachment of a stimulation node (Fig. 1S A) to the side of the WR using part Fig. 1S B.2. The interface for cylindrical surfaces can remain in place once it has been installed as it does not influence the WR.

**4. Results of kinematic impact**

| **Individuals** | **Variables**  **[°]** | **Stimulation conditions M (SD)** | | | | **Repeated measures ANOVA / *Friedman Test*** | | | | |
| --- | --- | --- | --- | --- | --- | --- | --- | --- | --- | --- |
| **SCI 1** |  | **No Stimulation (C1)** | **NP TA GS H Standard (C2)** | **NP TA H Standard (C3)** |  | **gl** | **F / *Chi*** | **p** | **η^2^** | **β-1** |
| Left side  (N = 21) | A1 | -8.20 (0.94) | -9.29 (1.95) | -6.51 (1.20) |  | *2* | *22.57* | *0.00* |  |  |
|  | A2 | 0.13 (1.07) | -0.03 (0.93) | 1.57 (1.06) |  | *2* | *20.67* | *0.00* |  |  |
|  | A3 | -5.81 (0.63) | -7.18 (1.36) | -5.60 (0.73) |  | 2 | 17.85 | 0.00 | 0.47 | 1.00 |
|  | K1 | 37.43 (2.31) | 33.54 (1.64) | 37.96 (2.32) |  | *2* | *20.67* | *0.00* |  |  |
| **SCI 2** |  | **No Stimulation (C1)** | **NP GS** **Standard (C2)** | **NP TA GS Standard (C3)** |  |  |  |  |  |  |
| Left side  (N = 29) | A1 | 3.43 (1.94) | -2.22 (2.90) | 0.01 (3.70) |  | *2* | *27.52* | *0.00* |  |  |
|  | A2 | -1.72 (2.07) | -4.98 (2.08) | -6.49 (2.06) |  | *2* | *33.17* | *0.00* |  |  |
|  | A3 | 5.91 (2.05) | -0.04 (1.83) | 2.65 (2.46) |  | *2* | *44.90* | *0.00* |  |  |
| Right side  (N = 24) | A1 | -1.80 (3.85) | -2.23 (3.75) | 1.30 (3.35) |  | 2 | 6.66 | 0.03 | 0.22 | 0.90 |
|  | A2 | 0.27 (2.97) | -1.48 (3.08) | -2.39 (2.80) |  | 2 | 5.15 | 0.01 | 0.18 | 0.80 |
|  | A3 | 8.95 (2.52) | 4.43 (2.93) | 5.61 (4.02) |  | 1.41 | 14.90 | 0.00 | 0.39 | 0.99 |
| **Stroke 1** |  | **No Stimulation (C1)** | **NP TA GS H Cross (C2)** | **NP TA GS H** **Standard (C3)** | **WH TA GS H Standard (C4)** |  |  |  |  |  |
| Left side  (N = 11) | A1 | -18.07 (3.37) | 6.57 (1.29) | 4.16 (6.29) | -13.06 (2.42) | 3 | 122.18 | 0.00 | 0.92 | 1.00 |
|  | A2 | -11.44 (4.51) | 1.29 (2.48) | 1.91 (2.78) | -6.81 (4.28) | 3 | 39.48 | 0.00 | 0.80 | 1.00 |
|  | A3 | -11.99 (1.68) | 9.14 (1.02) | 9.72 (1.81) | -7.08 (5.41) | *3* | *8.13* | *0.04* |  |  |
|  | K1 | 39.92 (5.09) | 41.36 (4.35) | 44.61 (4.34) | 43.86 (0.19) | 3 | 3.20 | 0.04 | 0.24 | 0.68 |
| **Stroke 2** |  | **No Stimulation (C1)** | **WR (C2)** | **WH TA GS H** **Standard (C3)** |  |  |  |  |  |  |
| Left side  (N = 6) | A1 | -10.18 (1.57) | -12.14 (2.48) | -8.03 (6.64) |  | *2* | *4.33* | *0.12* |  |  |
|  | A2 | -15.57 (2.53) | -7.53 (3.99) | -7.09 (2.99) |  | *2* | *12.00* | *0.00* |  |  |
|  | A3 | -9.84 (1.55) | -4.79 (1.96) | 0.76 (1.17) |  | 2 | 84.76 | 0.00 | 0.94 | 1.00 |
|  | K1 | 32.89 (0.85) | 51.35 (0.60) | 51.77 (0.28) |  | 2 | 1857.87 | 0.00 | 0.99 | 1.00 |

Table 2S. Kinematic impact results. This table shows the means (M) and deviations (SD) of the study variables: maximum ankle dorsiflexion at heel-contact (A1), plantarflexion angle at toe-off (A2), maximum dorsiflexion during the mid-swing (A3) and maximum knee flexion during the swing (K1). The number (N) of gait cycles that could be recorded per individual and side is shown. The results of the repeated measures ANOVA or Friedman's test are also shown according to whether or not the normality condition was satisfied. The results of the Friedman test are shown in italics. For the repeated-measures ANOVA results, the effect size and statistical power were also calculated.

| **Individuals** | **Variables**  **[°]** | **Pair comparisons** | **Left** | **Right** |
| --- | --- | --- | --- | --- |
|  |  |  | **p-value [** **IC 95% Lower limit,** **Upper limit]** | **p-value [** **IC 95% Lower limit, Upper limit]** |
| **SCI 1** | A1 | C1 vs C2 | 0.050 |  |
|  |  | C1 vs C3 | **0.000** |  |
|  |  | C2 vs C3 | **0.000** |  |
|  | A2 | C1 vs C2 | 0.357 |  |
|  |  | C1 vs C3 | **0.001** |  |
|  |  | C2 vs C3 | **0.000** |  |
|  | A3 | C1 vs C2 | **0.001 [0.59 2.15]** |  |
|  |  | C1 vs C3 | 1.000 [-0.78 0.35] |  |
|  |  | C2 vs C3 | **0.000 [-2.45 -0.72]** |  |
|  | K1 | C1 vs C2 | **0.000** |  |
|  |  | C1 vs C3 | 0.961 |  |
|  |  | C2 vs C3 | **0.000** |  |
| **SCI 2** | A1 | C1 vs C2 | **0.000** | 1.000 [-2.18 3.04] |
|  |  | C1 vs C3 | **0.000** | **0.035 [-6.03 -0.17]** |
|  |  | C2 vs C3 | **0.004** | **0.006 [-6.17 -0.90]** |
|  | A2 | C1 vs C2 | **0.000** | 0.150 [-0.44 3.94] |
|  |  | C1 vs C3 | **0.000** | **0.003 [0.82 4.50]** |
|  |  | C2 vs C3 | **0.016** | 1.000 [-1.55 3.36] |
|  | A1 | C1 vs C2 | **0.000** | **0.000 [3.09 5.97]** |
|  |  | C1 vs C3 | **0.000** | **0.003 [1.08 5.61]** |
|  |  | C2 vs C3 | **0.000** | 0.840 [-3.94 1.58] |

Table 3S. Pairwise comparison. The results of the pairwise comparison between conditions for SCI 1 and SCI 2 individuals are shown for each of the variables and sides measured. In the repeated measures ANOVA, the significance and confidence interval are provided; in the case of the Friedman test, only the significance is provided. Statistically significant results are highlighted in bold.

| **Individual** | **Variables**  **[°]** | **Pair comparisons** | **Left side** | **Individual** | **Variables** | **Pair comparisons** | **Left side** |
| --- | --- | --- | --- | --- | --- | --- | --- |
|  |  |  | **p-value [** **IC 95% Lower limit,** **Upper limit]** |  |  |  | **p-value [** **IC 95% Lower limit,** **Upper limit]** |
| **Stroke 1** | A1 | C1 vs C2 | **0.000 [-28.18 -21.09]** | **Stroke 2** | A1 | C1 vs C2 | 0.249 |
|  |  | C1 vs C3 | **0.000 [-28.69 -15.77]** |  |  | C1 vs C3 | 0.345 |
|  |  | C1 vs C4 | **0.011 [-8.91 -1.10]** |  |  | C2 vs C3 | 0.075 |
|  |  | C2 vs C3 | 1.000 [-3.95 8.77] |  | A2 | C1 vs C2 | **0.028** |
|  |  | C2 vs C4 | **0.000 [16.73 22.54]** |  |  | C1 vs C3 | **0.028** |
|  |  | C3 vs C4 | **0.000 [10.82 23.63]** |  |  | C2 vs C3 | 0.917 |
|  | A2 | C1 vs C2 | **0.000 [-17.16 -8.32]** |  | A3 | C1 vs C2 | **0.008 [-8.30 -1.80]** |
|  |  | C1 vs C3 | **0.000 [18.10 -8.62]** |  |  | C1 vs C3 | **0.000[-13.38 -7.81]** |
|  |  | C1 vs C4 | 0.090 [-9.82 0.55] |  |  | C2 vs C3 | **0.002 [-8.11 -3.00]** |
|  |  | C2 vs C3 | 1.000 [-4.42 3.19] |  | K1 | C1 vs C2 | **0.000 [-20.05 -16.88]** |
|  |  | C2 vs C4 | **0.002 [3.12 13.09]** |  |  | C1 vs C3 | **0.000 [-20.12 -17.63]** |
|  |  | C3 vs C4 | **0.002 [3.34 14.10]** |  |  | C2 vs C3 | 0.372 [-1.20 0.38] |
|  | A3 | C1 vs C2 | **0.000** |  |  |  |  |
|  |  | C1 vs C3 | **0.003** |  |  |  |  |
|  |  | C1 vs C4 | **0.001** |  |  |  |  |
|  |  | C2 vs C3 | 0.075 |  |  |  |  |
|  |  | C2 vs C4 | **0.000** |  |  |  |  |
|  |  | C3 vs C4 | **0.003** |  |  |  |  |
|  | K1 | C1 vs C2 | 1.000 [-7.70 4.83] |  |  |  |  |
|  |  | C1 vs C3 | 0.390 [-12.10 2.72] |  |  |  |  |
|  |  | C1 vs C4 | 0.156 [-8.90 1.01] |  |  |  |  |
|  |  | C2 vs C3 | 0.635 [-9.26 2.74] |  |  |  |  |
|  |  | C2 vs C4 | 0.483 [-6.75 1.72] |  |  |  |  |
|  |  | C3 vs C4 | 1.000 [-3.61 5.10] |  |  |  |  |

Table 4S. Pairwise comparison. The results of the pairwise comparison between conditions for Stroke 1 and Stroke 2 individuals are shown for each of the variables and left side measured. In the repeated measures ANOVA, the significance and confidence interval are provided; in the case of the Friedman test, only the significance is provided. Statistically significant results are highlighted in bold.


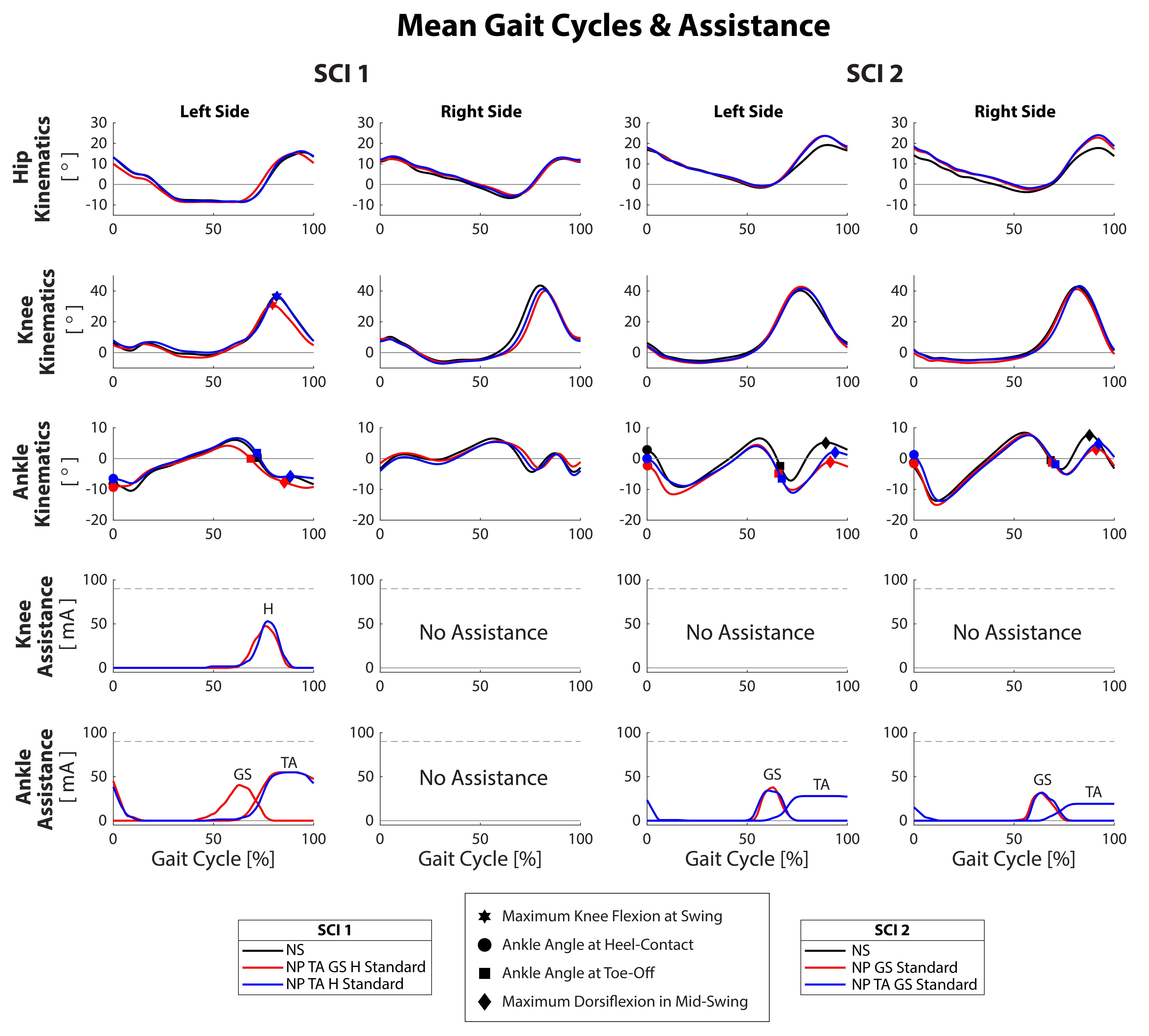


Fig. 10S. Mean gait cycles and assistance. This figure shows the mean gait cycles of individuals SCI 1 and SCI 2. The mean gait cycles are shown for each of the configurations and the variables that were measured: maximum ankle dorsiflexion at heel-contact (A1), ankle angle at toe-off (A2), maximum dorsiflexion during the mid-swing (A3) and maximum knee flexion during the swing (K1). The average assistance received by each individual in each muscle group according to the established configuration is also shown: hamstrings (H), gastrocnemius (GS) and tibialis anterior (TA).


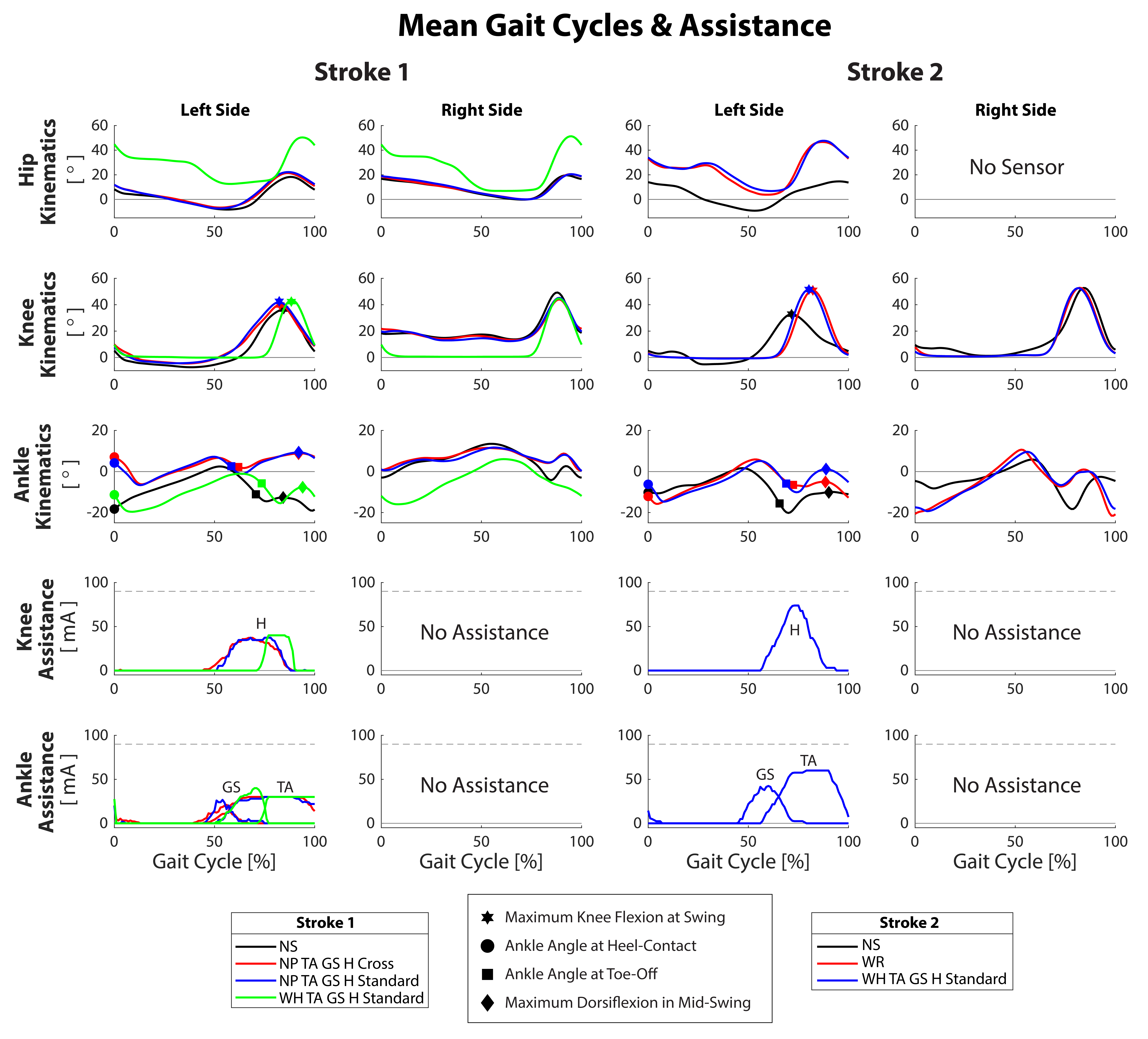


Fig. 11S. Mean gait cycles and assistance. This figure shows the mean gait cycles of individuals Stroke 1 and Stroke 2. The mean gait cycles are shown for each of the configurations and the variables that were measured: maximum ankle dorsiflexion at heel-contact (A1), ankle angle at toe-off (A2), maximum dorsiflexion during the mid-swing (A3) and maximum knee flexion during the swing (K1). The average assistance received by each individual in each muscle group according to the established configuration is also shown: hamstrings (H), gastrocnemius (GS) and tibialis anterior (TA). No kinematic data are shown for the right hip of stroke 2 due to sensor malfunctions, so that joint could not be recorded. The increase in overall flexion shown at the hip level in both individuals is observed in the configurations that include the use of the WR and is due to the forward leaning posture of the individuals when performing the gait activity.

**5. References**

1. Ltd B. Equipment for DAQ Research & Clinical Rehab | Biometrics Ltd [Internet]. [cited 2024 Jan 4]. Available from: https://www.biometricsltd.com/index.htm

2. Abacus. Power Bank DCU 10.000 mAh Dual output - Abacus online [Internet]. [cited 2024 Jan 8]. Available from: https://www.abacus.coop/es/power-bank-dcu-10.000-mah-dual-output/1379968.68.html

3. Brunetti F, Garay Á, Moreno JC, Pons JL. Enhancing functional electrical stimulation for emerging rehabilitation robotics in the framework of hyper project. In: IEEE International Conference on Rehabilitation Robotics. 2011. p. 1–13.

4. Souza DC de, Gaiotto M do C, Nogueira Neto GN, Castro MCF de, Nohama P. Power amplifier circuits for functional electrical stimulation systems. Res Biomed Eng. 2017;33(2):144–55.

5. Loreiro M, Britez S, Casco S, Moreno JC, Pons JL, Brunetti F. Neuroprosthetic device for functional training, compensation or rehabilitation of lower limbs during gait. In: International IEEE/EMBS Conference on Neural Engineering, NER. 2019. p. 1183–6.

6. Khan A, Li K, Wei N. Integrated design of functional electrical stimulator and transcutaneous electrical nerve stimulator on a single prototype. In: 2021 6th IEEE International Conference on Advanced Robotics and Mechatronics, ICARM 2021. 2021. p. 453–8.

7. Electronics M. XW1E-BV404M-R IDEC:Mouser [Internet]. [cited 2024 Jan 5]. Available from: https://www.mouser.es/ProductDetail/199-XW1EBV404MR

8. Loeb GE, Peck RA, Moore WH, Hood K. BION^TM^ system for distributed neural prosthetic interfaces. Med Eng Phys. 2001;23(1):9–18.

9. Weber DJ, Stein RB, Chan KM, Loeb GE, Richmond FJR, Rolf R, et al. Functional electrical stimulation using microstimulators to correct foot drop: A case study. Can J Physiol Pharmacol. 2004;82(8–9):784–92.

10. Popovic MR, Keller T. Modular transcutaneous functional electrical stimulation system. Med Eng Phys. 2005;27(1):81–92.

11. Qu H, Xie Y, Liu X, He X, Hao M, Bao Y, et al. Development of network-based multichannel neuromuscular electrical stimulation system for stroke rehabilitation. J Rehabil Res Dev. 2016;53(2):263–78.

12. Qu H, Wang T, Hao M, Shi P, Zhang W, Wang G, et al. Development of a network FES system for stroke rehabilitation. In: Proceedings of the Annual International Conference of the IEEE Engineering in Medicine and Biology Society, EMBS. IEEE; 2011. p. 3119–22.

13. Jovičić NS, Saranovac L V., Popović DB. Wireless distributed functional electrical stimulation system. J Neuroeng Rehabil. 2012;9(1):1–10.

14. Andreu D, Sijobert B, Toussaint M, Fattal C, Azevedo-Coste C, Guiraud D. Wireless electrical stimulators and sensors network for closed loop control in rehabilitation. Front Neurosci. 2020;14(117):1–12.

15. Cerone GL, Giangrande A, Vieira T, Pisaturo D, Ionescu M, Gazzoni M, et al. Design of a programmable and modular neuromuscular electrical stimulator integrated into a wireless body sensor network. IEEE Access. 2021;9:163284–96.

16. Kilgore KL, Smith B, Campean A, Hart RL, Lambrecht JM, Buckett JR, et al. Powering strategies for implanted multi-function neuroprostheses for spinal cord injury. Healthc Technol Lett. 2020;7(3):81–6.

17. Makowski N, Campean A, Lambrecht J, Buckett J, Coburn J, Hart R, et al. Design and Testing of Stimulation and Myoelectric Recording Modules in an Implanted Distributed Neuroprosthetic System. IEEE Trans Biomed Circuits Syst. 2021;15(2):281–93.
